# Effectiveness of a group psychological intervention to reduce psychosocial distress in adolescents in Pakistan: a single-blind, cluster randomised controlled trial

**DOI:** 10.1016/S2352-4642(24)00101-9

**Published:** 2024-08

**Authors:** Syed Usman Hamdani, Zill-e Huma, Aiysha Malik, Asad Tamizuddin-Nizami, Hashim Javed, Fareed Aslam Minhas, Mark J D Jordans, Marit Sijbrandij, Nadia Suleman, Um-ul Baneen, Richard Allan Bryant, Mark van Ommeren, Atif Rahman, Duolao Wang

**Affiliations:** a Global Institute of Human Development, Shifa Tameer-e-Millat University, Islamabad, Pakistan; b Department of Mental Health and Substance Use, WHO, Geneva, Switzerland; c Institute of Psychiatry, Benazir Bhutto Hospital, Rawalpindi Medical University, Rawalpindi, Pakistan; d Amsterdam Institute of Social Science Research, University of Amsterdam, Amsterdam, Netherlands; e School of Psychology, University of New South Wales, Sydney, NSW, Australia; f Amsterdam Public Health Research Institute, Vrije Universiteit Amsterdam, Amsterdam, Netherlands; g Department of Primary Care and Mental Health, University of Liverpool, Liverpool, UK; h Global Health Trials Unit, Liverpool School of Tropical Medicine, Liverpool, UK

## Abstract

**Background:**

Emotional problems in adolescents living in low-income and middle-income countries (LMICs) remain largely unaddressed; key reasons include a scarcity of trained mental health professionals and unavailability of evidence-based, scalable psychological interventions. We aimed to evaluate the effectiveness of a non-specialist-delivered, group psychological intervention to reduce psychosocial distress in school-going adolescents in Pakistan.

**Methods:**

In a two-arm, single-blind, cluster randomised controlled trial, eligible public school clusters from a rural subdistrict of Gujar Khan, Rawalpindi, Pakistan, were randomised (1:1, stratified by sex) using permuted block randomisation into intervention (n=20) and wait-list control (n=20) groups. Adolescents aged 13–15 years who provided informed assent and caregivers’ consent were screened for psychosocial distress using the youth-reported Pediatric Symptoms Checklist (PSC; total psychosocial distress scores from 0 to 70), and those scoring 28 or more and their caregivers were enrolled into the trial. Adolescents in the intervention group received seven weekly group sessions and their caregivers received three biweekly group sessions in school settings from trained non-specialists. The primary outcome was change from baseline in the total PSC scores at 3 months post-intervention. The trial was registered prospectively with the International Standard Randomised Controlled Trial Number registry, ISRCTN17755448.

**Findings:**

From the 40 school clusters that were included, 282 adolescents in the intervention group and 284 adolescents in the wait-list control group were enrolled between Nov 2 and Nov 30, 2021. At 3 months, adolescents in the intervention group had significantly lower mean total score on the PSC compared with adolescents in the control group (mean difference in change from baseline 3·48 [95% CI 1·66–5·29], p=0·0002, effect size 0·38 [95% CI 0·18–0·57]; adjusted mean difference 3·26 (95% CI 1·46–5·06], p=0·0004, effect size 0·35 (0·16–0·55). No adverse events were reported in either group.

**Interpretation:**

The group psychological intervention most likely represents a feasible and effective option for adolescents with psychosocial distress in school settings.

**Funding:**

UK Medical Research Council, Foreign Commonwealth and Development Office, Department of Health and Social Care.

**Translation:**

For the Urdu translation of the abstract see Supplementary Materials section.

## Introduction

Common mental disorders are leading contributors to the burden of disease and health-related disability in children and adolescents globally.^1^ Untreated mental illness can adversely affect adolescents’ development, family and social life, and academic achievement. Adolescents living in low-income and middle-income countries (LMICs), which account for 90% of the world's population,^2^ are disproportionately affected by common mental disorders,^3^ probably due to the co-occurrence of multiple risk factors such as poverty, violence, and political instability, and insufficient mental health care.^4^ Two approaches have been advocated to overcome the gap between the need for and availability of mental health services for common mental disorders in LMICs: development of transdiagnostic mental health interventions that are designed to address a range of symptoms and do not require an expert diagnosis of a mental health condition to qualify treatment; and use of task-shifting that involves the delivery of mental health services by trained and supervised non-specialists.^5^

Schools are uniquely placed to reach substantial numbers of young people to address their mental health needs.^5^, ^6^ Systematic reviews and meta-analyses have provided strong evidence for the effectiveness of school-based psychosocial programmes, including improvement in mental health, social emotional competence, and academic outcomes, and reduction in negative health and social behaviours,^7^, ^8^, ^9^, ^10^ especially when delivered by non-specialists in LMICs.^11^ Although empirical evidence on school-based psychosocial interventions and emotional skills programmes has grown substantially, most evidence on effectiveness comes from high-income countries (HICs) and transferability to settings in LMICs is in a formative phase.^12^, ^13^ Culturally responsive school-based mental health interventions and context-specific research are considered integral to efforts that aim to address the mental health needs of adolescents in LMICs.^14^, ^15^, ^16^

**Research in context**

**Evidence before this study**

A sharp increase in the rates of anxiety and depression in adolescents puts many in need of psychological support in low-resource settings globally. Although a substantial number of school-based mental health interventions exist, the treatment effect of these interventions varies greatly in both high-income countries (HICs) and low-income and middle-income countries (LMICs). Zhang and colleagues (2023) did an updated meta-analysis of randomised controlled trials (RCTs) targeting the reduction in depression and anxiety in school-going adolescents in HICs. Their search yielded 29 RCTs published in English up to June, 2021, consisting of cognitive behavioural therapy (CBT) interventions (n=22) and non-CBT interventions (n=10) delivered by teachers or clinicians in HICs. The estimated weighted mean effect size for anxiety-related outcomes was 0·44 (p=0·001), whereas no statistically significant mean effect size for depression-related outcomes was observed (0·04, p=0·723). Similarly, Grande and colleagues (2023) did a meta-analysis of RCTs and non-RCTs published between 2007 and 2022 to assess the efficacy of school-based interventions addressing mental health concerns in children and adolescents in LMICs. The study included 39 school-based studies of various interventions, such as CBT (n=12), psychoeducation (n=13), neurocognitive approaches (n=5), narrative therapy (n=7), and meditation (n=4). The majority of these interventions were administered by lay health facilitators (n=30). The findings revealed no statistically significant treatment effect for anxiety (standardised mean difference 0·11; 95% CI –0·13 to 0·36) or depression (0·80; –0·47 to 2·07). Schools are an important public health platform, serving diverse age groups of children and adolescents and promoting both their academic and their mental wellbeing outcomes. School-based mental health programmes enable early identification and cost-effective management of mental health problems in adolescents in low-resource settings. Although school-based interventions show promise in addressing anxiety and depression among adolescents, their effectiveness varies across HICs and LMICs.

**Added value of this study**

Our cluster randomised controlled trial shows that a brief, multicomponent, CBT-based, non-specialist-led, group psychological intervention—Early Adolescent Skills for Emotions (EASE)—delivered under the supervision of country supervisors in school settings, was effective in reducing psychosocial distress in adolescents studying in low-resource public schools in Pakistan. The study, which had an adequate sample size and power, and a high response rate 3 months after the intervention, showed strong intervention effects in reducing psychosocial distress in adolescents living in low-resource settings (effect size 0·39, 95% CI 0·59–0·18).

**Implications of all the available evidence**

Given growing interest in the use of school settings to enhance mental health care for at-risk adolescents through various delivery models, the findings of our study show that non-specialists, supervised by local experts, can effectively mitigate psychosocial distress among adolescents in Pakistan's low-resource public schools. Previous trials of the EASE intervention that showed a beneficial effect on measures of psychosocial distress add weight to the validity and generalisability of the study's findings.

35% of the Pakistani population are younger than 14 years. Adversities such as economic disparities, violence, and political instability make children susceptible to the development of early-onset mental health issues.^4^, ^17^ Pre-pandemic prevalence of psychosocial distress among school-going adolescents in rural Pakistan was 25%.^18^ In response to the need for mental health interventions for young adolescents, WHO developed Early Adolescent Skills for Emotions (EASE), a brief, transdiagnostic, group psychological intervention designed to be delivered by trained and supervised non-specialists to help young people and their caregivers with internalising problems such as symptoms of distress, depression, and anxiety.^19^ EASE has been translated and culturally adapted for implementation in areas affected by adversity.^20^, ^21^, ^22^, ^23^, ^24^ Field testing indicated that EASE is acceptable to adolescents with psychological distress and their caregivers in Jordan^20^ and Lebanon,^22^, ^25^ as well as to adolescents in a refugee camp for Burundi refugees and their caregivers in Tanzania.^24^ EASE was also effective compared with enhanced usual care in reducing internalising symptoms in Syrian refugee adolescents in Jordan.^26^

We aimed to evaluate the effectiveness of the culturally adapted EASE intervention to reduce psychosocial distress in school-going adolescents in the rural subdistrict of Gujar Khan in Rawalpindi, Pakistan, where the most commonly spoken language is Punjabi and the literacy level is 80%.^27^ With an agrarian economy, the subdistrict has 497 public schools (89 middle schools and 85 high schools), of which 231 are boys’ schools and 266 girls’ schools.

## Methods

### Study design

This two-arm, single-blind, wait-list controlled, cluster randomised controlled trial (RCT) was conducted in boys’ and girls’ public schools in the subdistrict of Gujar Khan, in which the spoken and taught language is Urdu. The study follows on from extensive needs assessment and stakeholder engagement^28^, ^29^ and an implementation feasibility study,^28^ in which we completed the translation and cultural adaptation of the intervention for implementation in public schools of Pakistan and established its feasibility for implementation.

Ethics approval was obtained from the Ethics Committee of the University of Liverpool, Liverpool, UK, the WHO Ethics Review Committee, and the Institutional Review Board of the Human Development Research Foundation, Islamabad, Pakistan. The gatekeeper consenting bodies for the schools are the School Education Department, Government of Punjab, Pakistan, and the District Education Department, Rawalpindi, Pakistan. Regulatory approvals were obtained as part of the feasibility study^28^ and before programme implementation from the School Education Department and the District Education Department. Cluster-level consent was obtained from school headteachers to ensure the participation of specific schools. The trial protocol has been published.^30^ The trial was registered prospectively with the ISRCTN registry on Oct 15, 2021, ISRCTN17755448.

### Participants

Trial participants were adolescents aged 13–15 years who were studying in public schools in Gujar Khan and screened positive for psychological distress on the Urdu version of the self-rated Pediatric Symptoms Checklist (PSC; range 0–70, validated cutoff score ≥28),^18^ and their caregivers. The paper-based PSC was administered to adolescents in classroom settings, and those who screened positive for psychosocial distress were evaluated against the eligibility criteria for trial participation. The baseline assessments with adolescents were done by trained assessment team members in school settings, whereas assessments with caregivers were completed at their home.

We excluded adolescents with deafness, blindness, and speech difficulties, or with a severe mental, neurological, or substance use disorder (eg, psychosis, mutism, intellectual disability, autism, or drug dependence) identified by the trained assessment team during screening.^30^ Adolescents with imminent risks were referred to a specialist mental health-care facility.

Written informed consent was obtained from headteachers and parents, and assent from adolescents of all participating schools.^30^

### Randomisation and masking

Each school was defined as a unit of randomisation. An independent researcher, who was not involved in the study, randomised the 40 eligible schools on a 1:1 allocation ratio using a permuted block randomisation method (block size of 4), stratified by sex, into intervention (n=20) and wait-list control (n=20) groups using computerised software. Allocation concealment was ensured by keeping random assignments in sequentially numbered sealed envelopes. It was not possible to mask adolescents, caregivers, intervention facilitators (school counsellors), intervention supervisors, data managers, or the trial manager to the treatment allocation because of the nature of the intervention. The assessors, trial statistician, and principal investigator were masked to treatment allocation status. To ensure masking during the trial, the intervention and assessment teams were based at separate office locations and did not have any interaction. The assessment team members were also non-residents of the study subdistrict, Gujar Khan. Furthermore, participants were each individually instructed not to disclose their treatment allocation status to the assessors during any follow-up assessments. The fidelity of masking was measured by having assessors guess the condition of each participant at the end of each assessment.

### Procedures

The EASE^19^ intervention was delivered to adolescent participants across seven weekly 90-min sessions. Adolescent sessions involved psychoeducation, problem solving, stress management, behavioural activation, and relapse prevention. EASE for caregivers was delivered over three 90-min group sessions in the third, fifth, and seventh weeks of the adolescent sessions. Caregiver sessions involved psychoeducation, active listening, quality time, praise, caregiver self-care, and relapse prevention. As participants were young adolescents and their parents generally had low levels of literacy, questions were read out by assessment team members who were trained to ask questions in a uniform and standardised way. Home practice of the EASE strategies was encouraged between sessions for both adolescents and caregivers.

Before the delivery of EASE in school settings, each school's headteacher, teachers, and school staff received an orientation session to explain trial procedures, trial timelines, assessment schedule (data collection timepoints), and interventional materials. Orientation sessions were also used to confirm venues for the intervention.^28^

Each EASE session was delivered by a primary facilitator and a cofacilitator who was specifically appointed for the duration of the trial. Facilitators were non-specialist school counsellors with graduate degrees in psychology, but otherwise had little or no previous experience of delivering psychological interventions.^31^ They were selected during a previous feasibility trial on the basis of intervention-specific competency acquired during weekly classroom training and case practice (at least one intervention group delivered by each facilitator). The competency was rated by trained supervisors, who were mental health specialists (a psychiatrist and a psychologist), in line with the apprenticeship model.^28^, ^30^, ^32^, ^33^ Supervision involved structured discussion of difficulties encountered in delivering EASE, management of adverse events, and self-care for the staff. Supervision also formed an integral part of continued training (eg, through role-play and associated teaching methods). The trained supervisors, responsible for supervising the delivery of EASE and ensuring intervention fidelity, received their training from a master trainer (developer of EASE) during the feasibility study.^31^

Intervention fidelity was assessed by supervisors in randomly selected, live observed sessions using an EASE session-specific checklist.^28^ The checklist also included items to assess the school counsellors’ group facilitation skills while delivering intervention sessions. Intervention fidelity was defined by the extent to which every intervention activity in each session was delivered and with what competency. Supervisors marked the status of every activity and whether it was completed or not, and rated the competency with which a school counsellor completed each activity on a three-point Likert scale (3=done well, 2=done partially, 1=needs to improve skill); competency scores below 2 on this scale prompted additional training and supervision for each intervention activity.

Mental health services are not available in public schools in Pakistan, so no structured programmes were delivered to adolescents in schools in the wait-list control group, who received usual care for the duration of the study. Participants in the wait-list control group were scheduled to receive EASE immediately after trial evaluation if the results of the study showed positive findings.

Teachers and students in public-sector schools are entitled to health care in public dispensaries and hospitals through an existing referral system. Results of the screening were shared with participants in both of the study groups by the research team. All trial participants, including adolescents and their caregivers, were free to seek specialist (psychologist or psychiatrist) support from the child and adolescent psychiatry department at the Institute of Psychiatry, Benazir Bhutto Hospital, Rawalpindi Medical University, Rawalpindi, Pakistan, the tertiary mental health-care facility of the region, as part of usual care, if needed. We maintained the record of health-care services sought by study participants in both study groups using an adapted version of the Client Service Receipt Inventory^28^ at 3-month follow-up.

### Outcomes

The primary outcome was change from baseline in the total psychosocial distress scores, as measured by the Urdu translation of the PSC,^34^ at 3 months post-intervention, in line with clinical guidelines.^35^ The youth version of the PSC has 35 items and 3 subscales: externalising, internalising, and attention problems. Items are rated on a three-point Likert scale (0=never, 1=sometimes, 2=often). The total score is calculated by summing the responses to all items and ranges from 0 to 70, in which the higher score indicates more distress. We chose the total psychosocial distress score cutoff of 28 or more based on a validation study of PSC with 5856 adolescents aged 13–15 years who were studying in 41 public schools in the adjoining subdistrict, Kallar Syedan, in Rawalpindi, Pakistan.^18^

Secondary outcomes were anxiety and depressive symptoms measured by the Revised Children's Anxiety and Depression Scale,^36^ depressive symptoms measured by the adolescent version of the Patient Health Questionnaire-9,^37^ wellbeing measured by the Short Warwick–Edinburgh Mental Well-being Scale,^38^ somatic complaints measured by the somatic symptoms checklist,^28^ child's quality of life measured by the Pediatric Quality of Life instrument,^39^ child's insight into their problems and wellbeing (ie, perceived psychological wellbeing) measured by the self-administered Psychological Outcome Profiles, children's version,^40^ problem solving measured by the Social Problem-Solving Inventory-Revised Short Form,^41^ perceived emotional support measured by the Perceived Emotional/Personal Support Scale,^42^ and experience of facing stigma measured by the Personal Rejection Subscale of the Pediatric Self-Stigmatization Scale.^43^ Caregiver-reported secondary outcomes were health-related quality of life, measured by the Pediatric Quality of Life Family Impact Module,^39^ and cost of health service utilisation measured by the Client Service Receipt Inventory.^44^ Parenting practices, measured by the Alabama Parenting Questionnaire, were reported by both adolescents and parents.^45^ All outcome measures were measured at baseline, immediately after treatment (1 week), and at 3 months post-intervention delivery in both groups (appendix 2 pp 3–4).

Information on severe adverse events, including death of a participant due to any cause, suicide attempt, hospital admission, and stigmatisation experiences, was collected at all follow-up timepoints. The cost-effectiveness of EASE, and details, psychometric properties, and results of the Strategy Use Questionnaire,^30^ administered at both child and caregiver levels at baseline, 1 week (immediate), and 3 months post-intervention, will be reported separately.

### Statistical analysis

Based on the findings of the previous prevalence study done in the same study settings,^18^ in which the standard PSC cutoff score of 28 or more gave a prevalence of psychosocial distress in adolescents of 25·5%, and on findings of other school-based mental health interventions using measures to assess psychosocial problems in children,^46^, ^47^ we assumed an effect size of 0·4 in the total scores of the PSC at 3-month post-intervention follow-up, and calculated that schools with about 150 adolescents in grades 8 and 9 were needed to fulfil our sample size requirement. Power calculations suggested a sample size of 550 adolescents (ie, 225 in each group) from 40 school clusters and about 14 adolescents from each school (power=0·80; α=0·05, two-sided), accounting for 20% attrition.

Primary data analyses were based on the intention-to-treat principle, with supplemental per-protocol analyses. Initial analyses compared baseline characteristics of participants between groups. Outcome measures were summarised at baseline and 3-month post-intervention follow-up by intervention group. For the primary outcome, crude and adjusted mean differences with 95% CIs between intervention and control at 3 months were derived from mixed models. A linear mixed model used treatment, visit, and interaction between treatment and visit as fixed effects, the baseline value of the PSC psychosocial distress score as a covariate, and participant and school cluster as random effects. Effect size was calculated using the difference in least square means between the intervention and control groups from mixed model analysis, divided by the pooled SD at each visit. The normality assumption for the primary outcome was visually assessed. In a sensitivity analysis, missing values for the data collected at 1-week (immediate) and 3-month post-intervention follow-up were imputed using the multiple imputation method. The adjusted linear mixed model was adjusted for prespecified covariates (ie, age, sex, and Patient Health Questionnaire-9 score), established at baseline. Subgroup analysis of the primary endpoint was done on the above prespecified covariates. Secondary outcomes were analysed in a similar way.

Binary outcomes were analysed in a generalised linear mixed model with treatment, visit, and interaction between treatment and visit as fixed effects, baseline measurement of the outcome variable as covariate, and participant and school cluster as random effects. Odds ratio (OR) with 95% CIs was derived for each visit. Descriptive statistics are shown for outcome variables and baseline characteristics by treatment group and visit. Continuous variables were summarised as number of observations, mean, standard deviation, minimum, and maximum, by treatment group and visit; categorical variables were summarised as number and percentage of research participants with mental health problems by treatment group and visit. Cronbach's alpha was calculated to determine the reliability of the Urdu versions of the instruments used in the trial.

### Role of the funding source

The funder of the study had no role in study design, data collection, data analysis, data interpretation, or writing of the report.

## Results

48 schools in Gujar Khan had 150 or more students within the required age bracket and were recommended by the District Education Department for enrolment in the trial. Of these 48 public schools, 40 middle schools and public high schools met the eligibility criteria (≥150 adolescents aged 13–15 years) for enrolment and were randomly assigned following stratified randomisation (figure).

**Figure:**
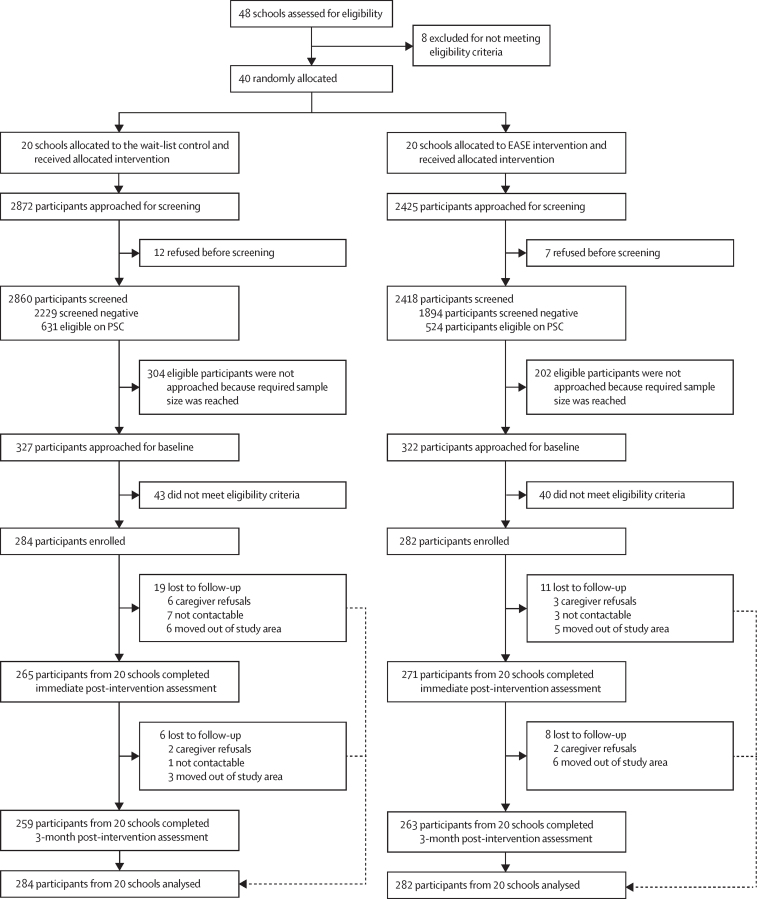
Trial profile EASE=Early Adolescent Skills for Emotions. PSC=Pediatric Symptoms Checklist.

649 adolescents were assessed for eligibility, and 566 (87%) met the eligibility criteria and, together with their caregivers, were enrolled in the study (mean cluster size of 14 [SD 3] adolescents per cluster) between Nov 2 and Nov 30, 2021. The mean age of adolescents was 13·87 (SD 0·75) years. Just over half of the participants were boys, most were living in a nuclear family, and most primary caregivers were women (table 1). The average monthly family income was below the minimum monthly wage in Pakistan (25 000 PKR).

**Table 1:** Demographic characteristics of study participants at baseline (intention-to-treat population)

		**All (n=566)**	**Wait-list control (n=284)**	**EASE intervention (n=282)**
Age, years		13·87 (0·75)	13·85 (0·75)	13·89 (0·75)
Sex				
	Male	294 (52%)	141 (50%)	153 (54%)
	Female	272 (48%)	143 (50%)	129 (45%)
Number of siblings		4·11 (1·61)	4·22 (1·48)	4·00 (1·74)
Family structure				
	Nuclear family system	327 (58%)	158 (56%)	169 (60%)
	Joint family system	239 (42%)	126 (44%)	113 (40%)
Mother's education, years		7·04 (4·28)	7·01 (4·22)	7·08 (4·35)
Father's education, years		5·59 (4·54)	5·48 (4·57)	5·70 (4·52)
Father's employment status*				
	Professional	10 (2%)	2 (1%)	8 (3%)
	Manual worker	229 (43%)	129 (49%)	100 (38%)
	Skilled worker	60 (11%)	33 (13%)	27 (10%)
	Government job	44 (8%)	24 (9%)	20 (8%)
	Personal business	75 (14%)	29 (11%)	46 (17%)
	Not working	60 (11%)	33 (13%)	27 (10%)
	Other	51 (10%)	14 (5%)	37 (14%)
Mother's employment status†				
	Professional	5 (1%)	1 (<1%)	4 (1%)
	Manual worker	34 (6%)	15 (5%)	19 (7%)
	Skilled worker	29 (5%)	20 (7%)	9 (3%)
	Private job	1 (<1%)	0	1 (<1%)
	Personal business	1 (<1%)	1 (<1%)	0
	Not in paid work	488 (87%)	242 (86%)	246 (88%)
	Other	1 (<1%)	1 (<1%)	0
Monthly income, PKR		23 963·20 (17 567·23)	24 746·27 (21 293·20)	23 147·67 (12 572·92)

Data are mean (SD) or n (%). EASE=Early Adolescent Skills for Emotions. PKR=Pakistani rupees.

* Because of missing data, father's employment status was available for 529 participants (264 in the wait-list control group and 265 in the EASE intervention group).

† Because of missing data, mother's employment status was available for 559 participants (280 in the wait-list control group and 279 in the EASE intervention group).

The Cronbach's alpha reliability of measurements ranged from 0·61 to 0·96. Mean time to administer the complete set of outcome measures at baseline and follow-up assessment was 43·09 (SD 29·71) min for adolescents and 23·41 (29·57) min for caregivers.

The intervention was delivered by 12 non-specialist facilitators in 20 intervention schools. 239 (84%) of 282 trial participants attended at least six of seven EASE sessions and 189 (67%) of 282 caregivers attended at least two of three caregiver sessions. Mean duration of adolescent group sessions was 103·08 (SD 19·63) min. Mean duration of caregiver group sessions was 84·65 (18·73) min.

Among the 522 (92%) of 566 trial participants for whom primary endpoint data at 3 months post-intervention were available for analysis (table 2), we found significant differences between intervention and control groups in mean PSC score change from baseline (primary outcome) and in mean score reductions on the PSC internalising, externalising, and attention subscales. The intervention and control groups did not differ in the mean scores of caregivers’ quality of life and parenting practices at 3 months post-intervention. Child-reported secondary outcomes are also shown in table 2.

**Table 2:** Summary statistics and results from mixed model analysis of child-reported and caregiver-reported outcomes (intention-to-treat population)

		**Descriptive statistics**		Mixed model analysis*		**Effect size (95% CI)**†
		Wait-list control (n=284)	EASE intervention (n=282)	Difference (95% CI)	p value	
**Primary outcome**						
PSC total score						
	Baseline	33·69 (4·89)	32·69 (4·34)	..	..	..
	1 week	−12·42 (8·58)	−14·85 (9·16)	2·74 (0·94 to 4·55)	0·0029	0·30 (0·10 to 0·50)
	3 months	−13·34 (8·98)	−16·38 (8·65)	3·48 (1·66 to 5·29)	0·0002	0·38 (0·18 to 0·57)
**Child-reported secondary outcomes**						
PSC internalising score						
	Baseline	5·37 (1·75)	5·1 (1·88)	..	..	..
	1 week	−2·22 (2·53)	−2·56 (2·48)	0·58 (0·19 to 0·98)	0·0042	0·27 (0·09 to 0·46)
	3 months	−2·53 (2·62)	−3·00 (2·38)	0·70 (0·30 to 1·10)	0·0007	0·34 (0·14 to 0·54)
PSC externalising score						
	Baseline	4·85 (2·37)	5·07 (2·32)	..	..	..
	1 week	−1·36 (2·50)	−2·10 (2·81)	0·57 (0·19 to 0·95)	0·0035	0·29 (0·09 to 0·48)
	3 months	−1·54 (2·57)	−2·25 (2·91)	0·61 (0·22 to 0·99)	0·0021	0·29 (0·11 to 0·47)
PSC attention score						
	Baseline	5·64 (1·69)	5·48 (1·83)	..	..	..
	1 week	−2·21 (2·39)	−2·54 (2·36)	0·44 (0·07 to 0·82)	0·0214	0·22 (0·03 to 0·42)
	3 months	−2·24 (2·42)	−2·79 (2·34)	0·68 (0·30 to 1·06)	0·0005	0·32 (0·14 to 0·50)
RCADS total score						
	Baseline	64·44 (12·97)	61·69 (12·92)	..	..	..
	1 week	−15·01 (17·26)	−17·09 (17·77)	4·31 (2·11 to 6·52)	0·0001	0·34 (0·17 to 0·52)
	3 months	−17·27 (15·06)	−20·98 (12·98)	5·82 (3·59 to 8·05)	<0·0001	0·52 (0·32 to 0·72)
RCADS anxiety score						
	Baseline	63·63 (12·52)	60·81 (12·76)	..	..	..
	1 week	−13·70 (16·45)	−15·48 (17·50)	4·09 (2·01 to 6·16)	0·0001	0·34 (0·17 to 0·52)
	3 months	−15·95 (14·39)	−19·19 (12·67)	5·47 (3·37 to 7·57)	<0·0001	0·52 (0·32 to 0·71)
RCADS depression score						
	Baseline	62 (13·46)	60·65 (12·22)	..	..	..
	1 week	−14·22 (17·70)	−16·45 (16·37)	3·35 (1·17 to 5·53)	0·0027	0·27 (0·09 to 0·45)
	3 months	−15·56 (15·16)	−19·60 (13·20)	4·91 (2·70 to 7·11)	<0·0001	0·45 (0·25 to 0·66)
Somatic symptoms checklist						
	Baseline	4·1 (3·23)	3·54 (2·71)	..	..	..
	1 week	−0·12 (3·74)	−0·59 (3·70)	0·92 (0·27 to 1·56)	0·0056	0·29 (0·09 to 0·49)
	3 months	−0·34 (3·83)	−1·23 (3·50)	1·27 (0·62 to 1·92)	0·0001	0·42 (0·20 to 0·63)
SPSI-R positive problem orientation						
	Baseline	7·85 (3·09)	7·34 (2·79)	..	..	..
	1 week	0·35 (3·97)	1·15 (3·55)	−0·29 (−0·81 to 0·24)	0·2828	−0·10 (−0·28 to 0·08)
	3 months	0·82 (4·07)	1·35 (3·84)	−0·02 (−0·55 to 0·51)	0·9362	−0·01 (−0·21 to 0·19)
SPSI-R negative problem orientation						
	Baseline	8·57 (4·59)	8·23 (3·9)	..	..	..
	1 week	−1·49 (4·59)	−2·35 (4·70)	1·15 (0·44 to 1·86)	0·0015	0·28 (0·11 to 0·45)
	3 months	−1·56 (5·02)	−2·44 (4·79)	1·08 0·36 to 1·79)	0·0032	0·26 (0·09 to 0·44)
SPSI-R rational problem solving						
	Baseline	7·37 (4·57)	6·83 (3·76)	..	..	..
	1 week	0·43 (5·15)	1·87 (4·69)	−1·09 (−1·82 to −0·35)	0·0038	−0·26 (−0·44 to −0·09)
	3 months	1·32 (5·64)	2·35 (5·30)	−0·67 (−1·42 to 0·07)	0·0764	−0·16 (−0·34 to 0·02)
SPSI-R impulsivity/carelessness problem-solving style						
	Baseline	5·47 (5·15)	5·51 (4·28)	..	..	..
	1 week	0·71 (5·63)	−0·77 (5·44)	1·47 (0·65 to 2·29)	0·0004	0·30 (0·13 to 0·47)
	3 months	0·63 (5·76)	−0·02 (5·80)	0·64 (−0·19 to 1·47)	0·1321	0·13 (−0·04 to 0·30)
SPSI-R avoidance problem-solving style						
	Baseline	5·52 (3·56)	6·1 (3·39)	..	..	..
	1 week	−0·50 (4·46)	−1·83 (4·45)	0·80 (0·21 to 1·38)	0·0081	0·23 (0·06 to 0·40)
	3 months	−0·64 (4·67)	−2·27 (4·45)	1·09 (0·49 to 1·68)	0·0004	0·32 (0·15 to 0·50)
PEPSS support from family						
	Baseline	15·86 (11·12)	18·17 (10·95)	..	..	..
	1 week	−1·36 (6·07)	0·35 (7·01)	−2·11 (−3·39 to −0·83)	0·0013	−0·19 (−0·31 to −0·08)
	3 months	−0·98 (6·55)	0·50 (6·84)	−1·78 (−3·07 to −0·50)	0·0067	−0·16 (−0·27 to −0·04)
PEPSS support from non-family adults						
	Baseline	8·2 (9·3)	6·92 (8·17)	..	..	..
	1 week	−1·78 (5·32)	−1·05 (4·56)	−0·43 (−1·37 to 0·50)	0·3640	−0·05 (−0·17 to 0·06)
	3 months	−1·75 (5·71)	−1·00 (4·95)	−0·46 (−1·40 to 0·49)	0·3405	−0·06 (−0·17 to 0·06)
PEPSS support from friends						
	Baseline	17·43 (12·85)	16·66 (11·12)	..	..	..
	1 week	−2·68 (8·00)	−1·38 (8·04)	−0·88 (−2·50 to 0·74)	0·2852	−0·07 (−0·21 to 0·06)
	3 months	−2·30 (9·16)	−1·17 (9·26)	−0·65 (−2·28 to 0·99)	0·4375	−0·05 (−0·18 to 0·08)
PHQ-9-A score						
	Baseline	7·28 (5·05)	6·4 (4·11)	..	..	..
	1 week	−1·44 (5·37)	−2·52 (4·39)	1·71 (0·97 to 2·45)	<0·0001	0·40 (0·23 to 0·57)
	3 months	−2·28 (5·48)	−3·53 (4·44)	1·77 (1·03 to 2·52)	<0·0001	0·44 (0·25 to 0·62)
SWEMWBS score						
	Baseline	18·3 (3·37)	18·87 (3·23)	..	..	..
	1 week	0·78 (4·04)	1·83 (4·27)	−1·48 (−2·22 to −0·74)	<0·0001	−0·39 (−0·59 to −0·20)
	3 months	1·21 (4·05)	2·24 (4·45)	−1·44 (−2·18 to −0·70)	0·0002	−0·38 (−0·58 to −0·19)
PedsQL total score						
	Baseline	75·58 (13)	75·25 (12·16)	..	..	..
	1 week	4·00 (13·69)	8·95 (13·67)	−4·86 (−7·27 to −2·45)	<0·0001	−0·36 (−0·54 to −0·18)
	3 months	5·80 (13·98)	12·91 (13·87)	−6·84 (−9·26 to −4·41)	<0·0001	−0·53 (−0·72 to −0·34)
PSYCHLOPS Kids						
	Baseline	6·98 (3·5)	6·36 (3·54)	..	..	..
	1 week	−0·84 (3·76)	−2·34 (4·20)	1·92 (1·21 to 2·62)	<0·0001	0·48 (0·31 to 0·66)
	3 months	−0·88 (3·96)	−2·92 (4·01)	2·43 (1·72 to 3·14)	<0·0001	0·64 (0·46 to 0·83)
APQ involvement						
	Baseline	39·73 (11·96)	42·35 (11·88)	..	..	..
	1 week	3·11 (12·04)	4·17 (12·96)	−2·50 (−5·32 to 0·32)	0·0820	−0·19 (−0·40 to 0·02)
	3 months	7·50 (13·12)	9·02 (14·24)	−2·78 (−5·61 to 0·05)	0·0546	−0·20 (−0·40 to 0·00)
APQ positive parenting						
	Baseline	18·24 (4·91)	18·56 (4·55)	..	..	..
	1 week	0·25 (4·88)	1·43 (4·87)	−1·33 (−2·24 to −0·41)	0·0045	−0·29 (−0·48 to −0·09)
	3 months	0·92 (4·96)	1·35 (5·16)	−0·55 (−1·47 to 0·37)	0·2383	−0·12 (−0·32 to 0·08)
APQ poor supervision						
	Baseline	19·88 (5·38)	20·06 (5·08)	..	..	..
	1 week	−1·73 (5·49)	−1·59 (5·56)	−0·16 (−1·11 to 0·79)	0·7430	−0·03 (−0·22 to 0·16)
	3 months	−3·07 (6·09)	−3·52 (5·95)	0·39 (−0·56 to 1·35)	0·4196	0·07 (−0·10 to 0·23)
APQ inconsistent discipline						
	Baseline	12·88 (3·35)	12·71 (3·26)	..	..	..
	1 week	0·77 (4·25)	0·44 (4·18)	0·45 (−0·13 to 1·03)	0·1266	0·13 (−0·04 to 0·30)
	3 months	1·24 (4·40)	0·29 (4·44)	1·07 (0·48 to 1·65)	0·0004	0·32 (0·14 to 0·49)
APQ corporal punishment						
	Baseline	5·55 (2·16)	5·77 (2)	..	..	..
	1 week	−0·45 (2·26)	−0·69 (2·44)	−0·03 (−0·46 to 0·40)	0·8944	−0·02 (−0·26 to 0·22)
	3 months	1·82 (3·05)	1·45 (3·14)	0·11 (−0·32 to 0·55)	0·6111	0·04 (−0·12 to 0·21)
**Caregiver-reported outcomes**						
PedsQL Family Impact						
	Baseline	92·54 (13·93)	91·29 (13·04)	..	..	..
	1 week	4·08 (12·22)	5·49 (12·44)	−0·27 (−1·31 to 0·77)	0·6131	−0·04 (−0·19 to 0·11)
	3 months	4·18 (13·27)	6·00 (12·23)	−0·82 (−1·87 to 0·23)	0·1274	−0·14 (−0·33 to 0·04)
APQ involvement						
	Baseline	26·68 (7·93)	26·15 (7·23)	..	..	..
	1 week	3·34 (7·91)	6·19 (6·74)	−2·73 (−4·05 to −1·41)	<0·0001	−0·37 (−0·56 to −0·19)
	3 months	3·72 (8·24)	6·02 (7·13)	−2·20 (−3·53 to −0·87)	0·0012	−0·31 (−0·49 to −0·12)
APQ positive parenting						
	Baseline	19·87 (4·83)	20·03 (4·31)	..	..	..
	1 week	1·35 (5·54)	2·27 (4·79)	−1·13 (−2·22 to −0·03)	0·0442	−0·24 (−0·47 to −0·01)
	3 months	1·38 (5·56)	1·94 (4·55)	−0·75 (−1·85 to 0·35)	0·1822	−0·16 (−0·39 to 0·07)
APQ poor supervision						
	Baseline	13·26 (3·59)	14·92 (4·33)	..	..	..
	1 week	−0·10 (3·78)	−2·20 (4·47)	0·96 (0·08 to 1·85)	0·0332	0·28 (0·02 to 0·53)
	3 months	−1·24 (3·95)	−2·85 (4·57)	0·43 (−0·46 to 1·32)	0·3456	0·12 (−0·13 to 0·38)
APQ inconsistent discipline						
	Baseline	13·43 (3·82)	13·8 (3·39)	..	..	..
	1 week	1·10 (5·02)	−7·34 (127·79)	0·28 (−0·69 to 1·15)	0·5335	0·07 (−0·16 to 0·31)
	3 months	1·07 (4·78)	−7·14 (129·80)	−0·15 (−1·03 to 0·72)	0·7316	−0·04 (−0·28 to 0·20)
APQ corporal punishment						
	Baseline	6·77 (2·58)	7·38 (2·68)	..	..	..
	1 week	0·02 (2·66)	−0·52 (3·08)	0·12 (−0·33 to 0·56)	0·6096	0·05 (−0·13 to 0·23)
	3 months	0·11 (2·77)	−0·45 (2·99)	0·13 (−0·32 to 0·58)	0·5665	0·05 (−0·13 to 0·24)

Data are mean (SD), unless otherwise specified. Change from baseline is given for 1-week (immediate) and 3-month post-intervention follow-up. EASE=Early Adolescent Skills for Emotions. PSC=Pediatric Symptoms Checklist. RCADS=Revised Children's Anxiety and Depression Scale. SPSI-R=Social Problem-Solving Inventory-Revised Short Form. PEPSS=Perceived Emotional/Personal Support Scale. PHQ-9-A=Patient Health Questionnaire-9, adolescent version. SWEMWBS=Short Warwick–Edinburgh Mental Well-being Scale. PedsQL=Pediatric Quality of Life. PSYCHLOPS Kids=Psychological Outcome Profiles, children's version. APQ=Alabama Parenting Questionnaire.

* Linear mixed model included treatment, visit, and interaction between treatment and visit as fixed effects; the baseline value of an outcome as covariates; and participant and school cluster as random effects.

† Effect size was calculated using the difference in least square means between the intervention and control arms from mixed model analysis, divided by the pooled SD at each visit.

The results remained consistent in the per-protocol analysis (appendix 2 pp 5–7). After controlling for age and severity on the PSC at baseline, covariate-adjusted analysis showed that significant differences between intervention and control groups in the mean scores of primary and secondary outcomes at 3-month post-intervention follow-up remained consistent (appendix 2 pp 8–12). The intracluster correlation coefficient for the primary endpoint was 0·047.

Visual inspections of residuals from the mixed model analyses of primary and secondary continuous outcomes did not show any serious violation of the normality assumption (appendix 2 p 2). Sensitivity analyses revealed that the estimated treatment effects in the primary and secondary outcomes were insensitive to missing values (appendix 2 pp 20–23).

The findings of mixed model analysis showed no significant difference between the intervention and control groups in mean total scores on the personal rejection subscale of the Pediatric Self-Stigmatization Scale (appendix 2 p 17). No adverse events were reported in either group.

Among 30 of 200 intervention sessions delivered by the 12 facilitators, fidelity was good—ie, all facilitators delivered the required components of each session. The mean competency score (on a scale of 1–3) for adolescent sessions was 21·64 (SD 3·04) and for caregiver sessions was 24·77 (SD 1·29). Over the 3-month duration of EASE intervention delivery (7-week delivery for each group), 12 weekly group supervision meetings of school counsellors were done by in-country supervisors.

18 (6%) of 282 caregivers in the intervention group and seven (2%) of 284 in the wait-list control group contacted a primary health-care physician, and 55 (19%) of 282 caregivers in the intervention group and 44 (15%) of 284 in the wait-list control group contacted a private doctor for their child's health-related issues during the 3-month follow-up period. 50 (18%) of 282 adolescents in the intervention group and 51 (18%) of 284 in the control group were prescribed medicines. Details on use of health services across the two groups at 3 months post-intervention for the services they used during the past 3 months are provided in appendix 2 (p 19).

After the primary outcome assessment at post-intervention follow-up, outcome assessors correctly guessed the allocation status of 43 (15%) of 282 participants in the intervention group and 37 (13%) of 284 participants in the wait-list control group, indicating that masking was successful.

## Discussion

This cluster RCT evaluated the effectiveness of a culturally adapted, transdiagnostic, multicomponent EASE intervention among adolescents in public schools in Pakistan. Compared with wait-list control (controlling for age and baseline severity of symptoms), we found that EASE reduced psychosocial distress, anxiety, and depression among adolescents immediately after the 7-week intervention and at 3-month follow-up, indicating the effectiveness of the transdiagnostic intervention. In addition to the reduction in symptoms scores, we found significant differences in subjective reports of wellbeing on a range of perceived psychological problems, indicating that the intervention created a meaningful change in the adolescents’ lives. These findings have important implications for both management of psychosocial symptoms in adolescents and promotion of adolescents’ wellbeing. The triangulation of findings with other EASE trials in which PSC has been used as a primary outcome measure, such as those done in Jordan^26^ and Lebanon,^25^ strengthens the validity and generalisability of the study's findings.

We did not find positive effects of intervention on caregiver outcomes such as quality of life and self-reported improvements in parenting practices. This lack of change could be attributed to the fact that the current study was not powered to detect an effect of intervention on caregiver outcomes, and to the low attendance rate of caregivers in EASE sessions, which is probably due to challenges faced by mothers (primary caregivers) in reaching training venues—eg, school location and transportation, competing demands on caregivers’ time, and caregivers’ own health problems. The importance of caregivers’ involvement in improving adolescent mental health outcomes is well reported in the literature.^48^ Bryant and colleagues^26^ reported a positive impact of the EASE intervention in reducing inconsistent discipline in parenting. There is a need to further adapt intervention delivery to address the practical issues concerning caregivers’ participation in the programme and to evaluate the effect of EASE on caregivers’ outcomes in a definitive trial. However, in our trial, a lack of improvement in caregiver outcomes did not impede positive youth outcomes. This finding is supported by the literature, in which adolescents favour little involvement of parents in treatment programmes due to concerns about confidentiality.^49^

This trial contributes to the existing evidence on the effectiveness of indicated school-based mental health interventions in low-resource settings.^11^, ^50^ A meta-analysis showed positive effects of clinician-led, school-based cognitive behavioural therapy (CBT) programmes in HICs in reducing depression and anxiety in adolescents.^50^ By contrast, another meta-analysis showed that indicated mental health interventions delivered by non-specialists in school settings in LMICs might not reduce symptoms of anxiety and depression in adolescents.^11^ The current study contributes to the existing literature by showing positive outcomes for a non-specialist-delivered, indicated, multicomponent, mental health intervention for at-risk adolescents in low-resource public schools in Pakistan.

In Pakistan, as in many LMICs, while health-care workers might have an academic qualification in psychology, clinical psychology, or other mental health practice, there is no formal certification mechanism to practise as a clinical psychologist. Such individuals are considered to be non-specialists, in contrast to mental health professionals or specialists in other countries who receive such formal certification after completing structured clinical training. The results of the current study should be interpreted within the framework of the task-shifting approach proposed in the EASE manual.^31^ Similarly, a trial of a brief, lay counsellor-delivered, transdiagnostic, problem-solving intervention in low-income schools in India showed a modest effect of the indicated intervention in improving psychosocial outcomes among adolescents at 6 weeks post-intervention.^14^ The intervention sustained beneficial effects on psychosocial problems and mental health symptoms in adolescents over the 12-month follow-up period.^51^ The findings of preliminary research on EASE showed its acceptability among adolescents experiencing psychological distress and their caregivers, both in refugee camps and in schools in Tanzania,^24^ as well as among Syrian refugees residing in urban areas in Jordan and Lebanon.^20^, ^22^ In another definitive trial,^26^ the effectiveness of the EASE intervention was shown in reducing internalising symptoms among at-risk refugee adolescents. These results contribute to the growing body of evidence supporting the efficacy and applicability of non-specialist-delivered, transdiagnostic, indicated, psychological interventions in improving adolescent wellbeing in LMICs.

A key strength of our study was the integration of the EASE trial within the public school system. Extensive engagement with stakeholders as part of the formative phase,^28^ validation of the primary outcome measure for the target population,^18^ and cultural adaptation of the intervention by involving young people and their caregivers ensured its relevance to the target population. A feasibility cluster RCT informed the design of this definitive trial.^28^ Involvement and support from the highest office of the President of Pakistan,^52^ fostering formal collaborations among key stakeholders, such as the Ministry of Health, School Education Department, national and international academic institutions, and implementation partners, contributed to the successful conduct of the trial.

The empirically supported EASE programme integrates psychoeducation, relaxation techniques, behavioural activation, problem-solving skills, social support, relapse prevention, and parental engagement.^13^ Although the results presented here show the effectiveness of the multicomponent intervention package, the trial was not powered to evaluate the mechanism of action of these components or to compare subgroups of adolescents to evaluate which component improved psychosocial distress for which group of adolescents. This is an important area for future research.

The findings have important implications from a public mental health perspective. Schools are a platform to reach a large number of children and adolescents and to deliver mental health interventions by non-specialist facilitators such as school counsellors,^6^ especially in low-resource settings.^53^ In the context of Pakistan's fragmented mental health-care system, EASE emerged as an early transdiagnostic, non-specialist-delivered intervention that can help to bridge the treatment gap for child and adolescent mental health in low-resource public school settings. However, school-based interventions should be coupled with efficient referral pathways to mental health services that cater to adolescents who do not respond to the intervention.

EASE is part of a series of low-intensity psychological interventions for implementation by non-specialists in low-resource settings globally. The scalability potential of the EASE intervention is evidenced by the feasibility of implementation and trials in the diverse settings of Tanzania, Lebanon, Jordan, and Pakistan. EASE is an indicated intervention in the WHO–UNICEF Helping Adolescents Thrive toolkit.^54^ WHO has now released EASE as an open access intervention for global dissemination.^55^ The scalability potential of this intervention can also be inferred from the latest meta-analysis of a similar WHO non-specialist-delivered transdiagnostic intervention for adults called Problem Management Plus, which has been implemented in 23 different trials across the globe since its dissemination by WHO.^56^ The ongoing evaluation of the cost-effectiveness of EASE implementation in Pakistan will further inform scale-up efforts in low-resource settings globally.

A strength of the current study was the high retention rate at endpoint follow-up assessment (91% in the wait-list control group and 93% in the intervention group), which can be attributed to delivery of the intervention in the structured environment of schools. Other strengths include validation of the primary outcome in school-going adolescents in Pakistan, cultural adaptation of EASE for implementation in school settings with the involvement of adolescents, feasibility evaluation of a culturally adapted intervention, and branding of EASE as an emotional skills training workshop for adolescents to avoid the stigma of implementing a mental health intervention for adolescents with psychosocial distress in school settings. Since, the study was done in the public schools of one of the largest subdistricts in Punjab—Gujar Khan, located in the Rawalpindi district (a population of about 1 000 000), whose sociodemographic characteristics are similar to other rural subdistricts—the findings of the study can be generalised to adolescents studying in public schools of Punjab province in Pakistan and other similar populations.

The study had several limitations. We used a wait-list control as a comparator. Some studies have shown a large placebo effect for enhanced usual care,^26^, ^57^ which creates a different type of bias—a large Hawthorne effect in the control group. Given the absence of school-based mental health services in public schools in Pakistan, we chose wait-list control to reflect the real-life context. Our results mean that we can make a case to policy makers that such interventions are beneficial and necessary. Although the use of a wait-list control design was informed by our past experience, the wait-list control has been criticised for potentially exaggerating the treatment effects of CBT interventions,^58^, ^59^ and these concerns should be considered when interpreting our findings. We have planned sequential, multiple assignment, randomised trials (SMARTs) to address concerns related to the use of a wait-list control.

We showed the effectiveness of the EASE intervention in reducing psychosocial distress in adolescents over a 3-month period. A long-term follow-up (6 months post-intervention) will be required to evaluate whether the positive treatment effects persist over an extended period. Here we did not measure whether a change in the psychosocial symptoms score of adolescents resulted in improved academic outcomes or not. Future studies could measure whether changes in psychosocial symptoms scores result in improved academic outcomes.

Lastly, there was 67% attendance of caregivers in the intervention sessions. Therefore, we cannot draw conclusions about the effects of caregivers’ strategies to reduce psychosocial distress in adolescents. Further fully powered randomised controlled trials are needed to evaluate the effect of the intervention on caregiver outcomes.

In conclusion, the findings of this definitive cluster RCT of an indicated, multicomponent, group psychological intervention delivered by non-specialist facilitators in public schools show that EASE can be effective in improving psychological outcomes in adolescents and can help to bridge mental health treatment gap in low-resource settings.

### Contributors

### Data sharing

The deidentified dataset generated and analysed during the current study will be available from the corresponding author on reasonable request after 2 years from trial publication (starting from 2025).

## Declaration of interests

We declare no competing interests.

## Supplementary Materials

Urdu translation of the abstract
