# Supplementary material for: Effectiveness of a group psychological intervention to reduce psychosocial distress in adolescents in Pakistan: a single-blind, cluster randomised controlled trial
Source: Lancet Child Adolesc Health. 2024 Aug;8(8):559–70. doi: 10.1016/S2352-4642(24)00101-9 (PMC11254783; doi:10.1016/S2352-4642(24)00101-9)
Supplement: Urdu translation of the abstract [file mmc1.pdf]

THE LANCET
Child & Adolescent Health

Supplementary appendix 1

This translation in Urdu was submitted by the authors and we reproduce it as supplied. It has not been peer reviewed. *The Lancet*'s editorial processes have only been applied to the original in English, which should serve as reference for this manuscript.

اردو میں یہ ترجمہ مصنفین کے ذریعہ پیش کیا گیا تھا اور ہم اس کی ہو بہو دوبارہ تخلیق کرتے ہیں۔ اس کا ہم مرتبہ ماہروں کے ذریعہ جائزہ نہیں لیا گیا ہے۔ *لانسیٹ* کے ادارتی عمل کا اطلاق صرف انگریزی کے اصل پر ہوا ہے، جو اس نسخے کے حوالہ کے طور پر کام کرے گا

Supplement to: Hamdani SU, Huma Z-e, Malik A, et al. Effectiveness of a group psychological intervention to reduce psychosocial distress in adolescents in Pakistan: a single-blind, cluster randomised controlled trial. *Lancet Child Adolesc Health* 2024; **8:** 559–70.

خلاصہ

پسِ منظر

ترقی پذیر ممالک میں زیادہ تر نوجوانوں کے نفسیاتی وذہنی مسائل حل نہیں ہوپاتے۔ اس کی وجہ تربیت یافتہ ماہرینِ نفسیات و ذہنی امراض اور تحقیق پہ مبنی ذہنی صحت کےایسے پروگرامز جنہیں بڑے پیمانے پر لاگو کیا جاسکے کی ،کمی ہے ۔ہماری اس تحقیق کا مقصد پاکستان میں اسکول میں پڑھنے والے نوجوانوں میں نفسیاتی وذہنی مسائل کو کم کرنے میں تربیت یافتہ معاونین (Non-specialist Facilitators) کےذریعے گروپس میں دیئےجانے والے ایک نفسیاتی صحت کے پروگرام کی افادیت کا جائزہ لینا تھا۔

طریقہ کار

ہم نے ایک سنگل بلائنڈڈ، کلسٹر رینڈمائزڈ کنٹرولڈ ٹرائل(single blinded, cluster Randomized Controlled Trial)کا تحقیقی طریقہ استعمال کیا۔ اس تحقیق میں ہم نے، راولپنڈی، پاکستان کے ایک دیہی سب ڈسٹرکٹ ،گوجر خان، سے منتخب کردہ سرکاری اسکولوں کو پرمیوٹڈ بلاک رینڈمائزیشن (permuted block randomization) کا استعمال کرتے ہوئےتجرباتی (n=20) اور ویٹ لسٹ کنٹرول (n=20) گروپوں میں (1:1، جنس کے لحاظ) تقسیم کیا ۔

ہم نے صرف ایسے13-15 سال کے نوجوانوں کو پروگرام میں شامل کیا جنھوں نے خود اور ان کے والدین نے اس پروگرام میں شامل ہونے کی رضامندی لکھ کر دی۔ یوتھ رپورٹڈ پیڈیاٹرک سمپٹمز چیک لسٹ (Youth Reported Pediatric Symptoms Checklist-PSC) کا استعمال کرتے ہوئے13-15 سال کے نوجوانوں میں سماجی اور جذباتی دباو کی نشاندہی کی گئی۔PSC پہ سماجی اور جذباتی دباو کا ٹوٹل اسکور 0-70 کے درمیان ہوتا ہے۔صرف ایسے نوجوانوں جن کا سماجی اور جذباتی دباو کا ٹوٹل اسکور 28یا اس سے زیادہ آیاانھیں اور ان کے والدین کو اس تحقیق میں شامل کیا گیا۔ تجرباتی گروپ میں شامل نوجوانوں نے تربیت یافتہ معاونین سے اسکول میں پروگرام کی سات ہفتہ وارنشستیں گروپ کی صورت میں لیں اور ان کے والدین تین دو ہفتہ وار گروپ نشستوں میں شامل ہو ئے ۔ اس تحقیق کا بنیادی مقصد پروگرام کے تین ماہ بعد PSC کے ٹوٹل سماجی اور جذباتی دباو کےاسکور میں آنے والی تبدیلی کا جائزہ لینا تھا۔ یہ ٹرائل انٹرنیشنل اسٹینڈرڈ رینڈمائزڈ کنٹرولڈ ٹرائل نمبر رجسٹری

(International Standard Randomised Controlled Trial Number registry ISRCTN17755448)

کے ساتھ پیشگی رجسٹر کیا گیا تھا۔

نتائج

نومبر 2، 2021، سے نومبر 30، 2021 کے درمیان،40 اسکولوں میں تجرباتی گروپ میں 282 نوجوانوں اور ویٹ لسٹ کنٹرول گروپ میں 284 نوجوانوں کو شامل کیا گیا۔ تین ماہ بعد، تجرباتی گروپ میں نوجوانوں کا PSC کے ذہنی اور سماجی دباو کے کل اسکور کا اوسط، ویٹ لسٹ کنٹرول گروپ کے نوجوانوں کے مقابلے میں انتہائی کم تھا (ابتدائی اسکور سےپروگرام کے تین ماہ بعد کے اسکور میں فرق 3.48 [95% CI 1.66 – 5.29]، p=0·0002، ایفکٹ سائز0·38، [95% CI 0·18–0·57]، adjusted mean 3·26 , difference [95% CI 1·46–5·06]، p=0·0004 ، ایفکٹ سائز 0·35 [95% CI 0·16–0·55]

کسی بھی گروپ میں پروگرام کے کوئی بھی مضر اثرات سامنے نہیں آئے۔

تشریح

ان نتا ئج کی بنیاد پہ یہ کہا جاسکتا ہے کہ گروپ کی صورت میں دیا جانے والا یہ نفسیاتی و ذہنی صحت کا پروگرام اسکول جانے والے ایسے نوجوانوں جوسماجی اور جذباتی دباو کا شکار ہیں کے لیے مؤثر اور موزوں انتخاب ہو سکتا ہے۔
