# Supplementary material for: Effectiveness of a group psychological intervention to reduce psychosocial distress in adolescents in Pakistan: a single-blind, cluster randomised controlled trial
Source: Lancet Child Adolesc Health. 2024 Aug;8(8):559–70. doi: 10.1016/S2352-4642(24)00101-9 (PMC11254783; doi:10.1016/S2352-4642(24)00101-9)
Supplement: Supplementary appendix 2 [file mmc2.pdf]

THE LANCET
Child & Adolescent Health

Supplementary appendix 2

This appendix formed part of the original submission and has been peer reviewed. We post it as supplied by the authors.

Supplement to: Hamdani SU, Huma Z-e, Malik A, et al. Effectiveness of a group psychological intervention to reduce psychosocial distress in adolescents in Pakistan: a single-blind, cluster randomised controlled trial. *Lancet Child Adolesc Health* 2024; **8:** 559–70.

Supplementary Appendix to the Manuscript

Table of Contents

Graph 1: Normal probability plot for conditional residuals from the mixed model analyses of primary and secondary outcomes.. 2

Table S2: Secondary outcome measures and schedule of assessments 3

Table S3.1: Summary statistics and results from mixed model analysis of primary and secondary child's outcomes (changes from baseline) (per protocol analysis).. 5

Table S4: Summary statistics and results from mixed model analysis of primary and secondary outcomes (changes from baseline): covariate adjusted analysis where age, cluster, PSC included as covariates (Intention to treat population)... 8

Table S4.1: Summary statistics and results from mixed model analysis of primary and secondary outcomes (changes from baseline): covariate adjusted analysis where age, cluster, PSC included as covariates (per protocol analysis)... 13

Table 5: Summary statistics and results from mixed model analysis of child reposted self-stigmatizing scale (Intention to treat population) 17

Table S5.1: Summary statistics and results from mixed model analysis of child reposted self-stigmatizing scale (per protocol analysis)... 18

Table S6. Education/Health services utilization across two arms 3-months post intervention. ... 19

Table S7a: Summary statistics and results from mixed model analysis of primary and secondary outcomes (changes from baseline): Sensitivity analysis (multiple imputation) – Intension to Treat (ITT) Population 20

Table S7b: Summary statistics and results from mixed model analysis of primary and secondary outcomes (changes from baseline): Sensitivity analysis (multiple imputation) – Per Protocol Analysis.. 24

Graph 1: Normal probability plot for conditional residuals from the mixed model analyses of primary and secondary outcomes

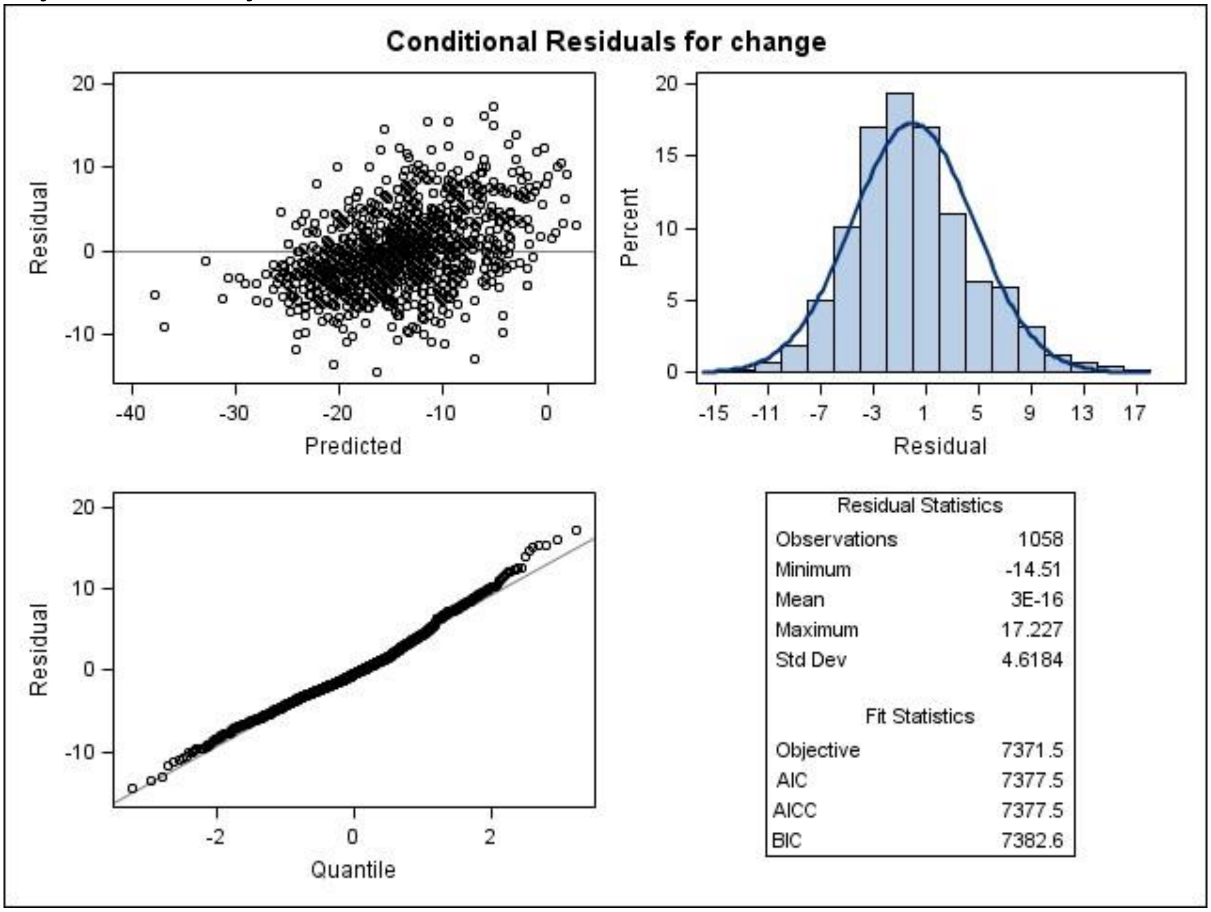

Table S2: Secondary outcome measures and schedule of assessments

	Description	Score	Baseline	1-week (immediate) post-intervention follow-up	3-month post-intervention follow-up
**Child-reported secondary outcome measures**					
Revised Children's Anxiety and Depression Scale (RCADS)[36]	25 items rated on a four-point scale (0=never, 1=sometimes, 2=often, 3=always)	Response values for each subscale are summed to give a total raw score (range 0-75); a higher score indicates more symptoms of anxiety and depression	Yes	Yes	Yes
Patient Health Questionnaire, adolescent version (PHQ-A)[37]	Nine items rated on a four-point Likert scale (0=not at all, 1=several days, 2=more than half the days, 3=nearly every day)	Responses to all items are summed to give a total score (range 0–27); a higher score indicates a higher incidence of depressive symptoms	Yes	Yes	Yes
Short Warwick–Edinburgh Mental Well-being Scale (SWEMWBS)[38]	Brief questionnaire designed to measure the mental wellbeing of adolescents over the past 2 weeks; seven items rated from "none of the time" to "all of the time"	The total score (range 7–35) is calculated by first summing the score for each of the seven items and then transforming the total raw scores to metric scores using the scale's conversion table; higher scores indicate higher levels of positive mental wellbeing	Yes	Yes	Yes
Somatic symptoms checklist[28]	Ten items rated on a three-point scale (0=not true, 1=somewhat true, 2=very true or often true)	The total score (range 0–20) is calculated by summing responses to all items; higher scores indicate frequent occurrence of somatic symptoms	Yes	Yes	Yes
Pediatric Quality of Life (PedsQL)[39]	23 items rated on a four-point scale (1=no problem to 4=almost always a problem)	Responses are linearly transformed to a 0–100 scale; higher scores indicate better quality of life	Yes	Yes	Yes
Psychological Outcome Profiles, children's version (PSYCHLOPS Kids)[40]	Assesses three domains ("problems, functioning, wellbeing) and has three questionnaire forms (pre-therapy, during therapy, and post-therapy versions); designed to be user-friendly and can be used by children as young as age 7 years	High scores (range 0–12) indicate poor functioning and wellbeing	Yes	Yes	Yes
Social Problem-Solving Inventory-Revised (SPSI-R) Short Form[41]	Five subscales, each with five items; two subscales (positive problem orientation and negative problem orientation) evaluate functional and dysfunctional cognitive and emotional orientations towards problem-solving; the remaining three subscales (rational problem solving, impulsivity/carelessness style, and avoidance style) assess problem-solving skills	Each subscale has a total score of 0–20; higher scores on positive and rational problem-solving orientations, and lower scores on negative problem-solving orientation, impulsivity, and avoidance style, indicate good social problem-solving	Yes	Yes	Yes
Perceived Emotional/Personal Support Scale (PEPSS)[42]	Designed to measure the level of emotional support adolescents receive from family, non-family adults, and friends; consists of four items; before responding to the main items, participants are first asked to list three important people for all three categories of relationships with whom they seek support	An emotional support score for each category of relationship is separately calculated by summing all items relevant to each category of relationship (range 4-12), where higher scores represent better social emotional support.	Yes	Yes	Yes

Personal rejection subscale of the Paediatric Self-Stigmatization Scale (PaedS)[43]	Five items rated on a four-point Likert scale (1=strongly disagree to 4=strongly agree), validated for its content and previously used in Pakistan[49]	Higher scores (range 5=20) indicate greater stigmatization	Yes	Yes	Yes
**Caregiver-reported secondary outcome measures**					
PedsQL Family Impact Module[39]	36-item scale that measures quality of life across six subscales (physical functioning, emotional functioning, social functioning, cognitive functioning, communication, and worry); caregivers report on family functioning via daily activities and family relationships; items are rated on a five-point Likert scale (0=never to 4=almost always)	The total score is calculated by summing all 36 items and dividing by the number of items answered, higher scores indicate better functioning (less negative impact)	Yes	Yes	Yes
Client Service Receipt Inventory[44]	Used to collect information on outpatient and inpatient services used by adolescents over a period of up to 9 months, including all types of health-care provider; records the number, location, and duration of services, as well as information on distance, cost, service quality, and sociodemographic characteristics	..	Yes	No	Yes
**Outcome reported by children and caregivers**					
Alabama Parenting Questionnaire (APQ)[45]	42 items rated on a five-point Likert scale (1=never to 5=almost always); covers five dimensions of parenting relevant to the aetiology and treatment of adolescents' problems, including positive involvement with children, supervision and monitoring, use of positive discipline techniques, consistency in the use of such discipline, and use of corporal punishment	Subscale scores range from 0 to 15 or 0 to 50, with higher scores indicating greater strength in the relevant subscale.	Yes	Yes	Yes

Table S3.1: Summary statistics and results from mixed model analysis of primary and secondary child's outcomes (changes from baseline) (per protocol analysis)

Visit	Descriptive statistics				Mixed model analysis		
	Wait-list control		EASE intervention		Difference (95%CI)	p-value	Effect Size
	n	Mean (SD)	n	Mean (SD)			
**PSC total score**							
1 week	265	-12.42(8.58)	266	-14.78(9.19)	2.65(0.85 to 4.46)	0.0040	0.29(0.09 to 0.49)
3 months	259	-13.34(8.98)	260	-16.39(8.67)	3.45(1.64 to 5.27)	0.0002	0.37(0.18 to 0.57)
**PSC internalizing score**							
1 week	265	-2.22(2.53)	266	-2.57(2.50)	0.57(0.17to 0.96)	0.0051	0.27(0.08 to 0.45)
3 months	259	-2.53(2.62)	260	-3.01(2.38)	0.70(0.30 to 1.10)	0.0007	0.34(0.15 to 0.54)
**PSC externalizing score**							
1 week	265	-1.36(2.50)	266	-2.11(2.83)	0.57(0.19 to 0.95)	0.0037	0.29(0.09 to 0.48)
3 months	259	-1.54(2.57)	260	-2.26(2.92)	0.62(0.23 to 1.00)	0.0019	0.29(0.11 to 0.48)
**PSC attention score**							
1 week	265	-2.21(2.39)	266	-2.53(2.37)	0.42(0.05 to 0.80)	0.0277	0.21(0.02 to 0.41)
3 months	259	-2.24(2.42)	260	-2.78(2.34)	0.67(0.28 to 1.05)	0.0006	0.31(0.13 to 0.49)
**RCADS total score**							
1 week	265	-15.01(17.26)	266	-17.09(17.82)	4.31(2.09 to 6.52)	0.0001	0.34(0.17 to 0.52)
3 months	259	-17.27(15.06)	260	-20.90(12.99)	5.76(3.53 to 7.99)	0.0001	0.52(0.32 to 0.72)
**RCADS anxiety score**							
1 week	265	-13.70(16.45)	266	-15.46(17.52)	4.09(2.01 to 6.18)	0.0001	0.35(0.17 to 0.52)
3 months	259	-15.95(14.39)	260	-19.08(12.66)	5.41(3.31 to 7.52)	0.0001	0.51(0.31 to 0.71)
**RCADS depression score**							
1 week	265	-14.22(17.70)	266	-16.50(16.48)	3.30(1.11 to 5.49)	0.0032	0.27(0.09 to 0.44)
3 months	259	-15.56(15.16)	260	-19.61(13.23)	4.87(2.66 to 7.08)	0.0001	0.45(0.25 to 0.65)
**Somatic symptoms**							
1 week	265	-0.12(3.74)	266	-0.61(3.71)	0.92(0.27 to1.57)	0.0056	0.29(0.09 to 0.49)
3 months	259	-0.34(3.83)	260	-1.23(3.52)	1.25(0.60 to 1.90)	0.0002	0.41(0.20 to 0.63)
****Positive problem orientation towards problem solving**							
1 week	265	0.35(3.97)	266	1.12(3.57)	-0.31(-0.84 to 0.22)	0.2548	-0.11(-0.29 to 0.08)
3 months	259	0.82(4.07)	260	1.28(3.78)	0.02(-0.52 to 0.55)	0.9563	0.01(-0.20 to 0.21)
****Negative problem orientation towards problem solving**							
1 week	265	-1.49(4.59)	266	-2.35(4.70)	1.13(0.42 to 1.84)	0.0018	0.28(0.10 to 0.45)
3 months	259	-1.56(5.02)	260	-2.46(4.81)	1.09(0.37 to 1.81)	0.0029	0.27(0.09 to 0.44)
****Rational orientation towards problem solving**							
1 week	265	0.43(5.15)	266	1.85(4.68)	-1.09(-1.84 to -0.34)	0.0045	-0.26(-0.45 to -0.08)
3 months	259	1.32(5.64)	260	2.25(5.25)	-0.61(-1.36 to 0.15)	0.1136	-0.14(-0.32 to 0.03)
****Impulsive/Carelessness style towards problem solving**							
1 week	265	0.71(5.63)	266	-0.82(5.47)	1.47(0.64 to 2.29)	0.0005	0.30(0.13 to 0.47)
3 months	259	0.63(5.76)	260	-0.04(5.81)	0.62(-0.21 to 1.46)	0.1414	0.13(-0.04 to 0.29)

Visit	Descriptive statistics				Mixed model analysis		
	Wait-list control		EASE intervention		Difference (95%CI)	p-value	Effect Size
	n	Mean (SD)	n	Mean (SD)			
****Avoidance problem solving style**							
1 week	265	-0.50(4.46)	266	-1.81(4.47)	0.74(0.15 to 1.33)	0.0135	0.21(0.04 to 0.38)
3 months	259	-0.64(4.67)	260	-2.29(4.47)	1.08(0.49 to 1.67)	0.0004	0.32(0.14 to 0.49)
**PEPSQ support from family**							
1 week	265	-1.36(6.07)	266	0.50(6.93)	-2.22(-3.48 to -0.96)	0.0006	-0.20(-0.32 to -0.09)
3 months	259	-0.98(6.55)	260	0.51(6.85)	-1.78(-3.04 to -0.51)	0.0060	-0.16(-0.27 to -0.054)
**PEPSQ support from non-family adults**							
1 week	265	-1.78(5.32)	266	-1.00(4.51)	-0.45(-1.39 to 0.48)	0.3406	-0.06(-0.17 to 0.06)
3 months	259	-1.75(5.71)	260	-0.96(4.95)	-0.51(-1.45 to 0.43)	0.2896	-0.06(-0.17 to 0.05)
**PEPSQ support from friends**							
1 week	265	-2.68(8.00)	266	-1.34(8.05)	-0.91(-2.53 to 0.72)	0.2735	-0.08(-0.21 to 0.06)
3 months	259	-2.30(9.16)	260	-1.13(9.29)	-0.71(-2.35 to 0.93)	0.3940	-0.06(-0.18 to 0.07)
**PHQ-9-A score**							
1 week	265	-1.44(5.37)	266	-2.56(4.42)	1.71(0.96 to 2.45)	0.0001	0.39(0.22 to 0.57)
3 months	259	-2.28(5.48)	260	-3.55(4.46)	1.77(1.02 to 2.52)	<0.0001	0.44(0.25 to 0.62)
**SWEMWS score**							
1 week	265	0.78 (4.04)	266	1.79(4.29)	-1.44 (-2.19 to -0.70)	0.0001	-0.38 (-0.58 to -0.19)
3 months	259	1.21 (4.05)	260	2.22 (4.45)	-1.41 (-2.16 to -0.66)	0.0002	-0.37 (-0.57 to -0.18)
**PedsQL total score**							
1 week	265	4.00(13.69)	266	8.97(13.79)	-4.77(-7.19 to -2.35)	0.0001	-0.36(-0.54 to - 0.18)
3 months	259	5.80(13.98)	260	12.97(13.91)	-6.80(-9.24 to -4.37)	0.0001	-0.53(-0.72 to -0.34)
**PedsQL physical functioning**							
1 week	265	3.18(18.74)	266	7.89(18.28)	-3.81(-6.72 to -0.89)	0.0107	-0.25(-0.43 to -0.06)
3 months	259	2.93(19.98)	260	10.85(18.43)	-6.57(-9.51 to -3.63)	0.0001	-0.40(-0.58 to -0.22)
**PedsQL emotional functioning**							
1 week	265	5.16(18.94)	266	11.08(19.67)	-6.14(-9.36 to - 2.91)	0.0002	-0.34(-0.51 to -0.16)
3 months	259	9.39(19.96)	260	16.20(21.26)	-7.08(-10.33 to -3.84)	.00001	-0.40(-0.59 to -0.22)
**PedsQL social functioning**							
1 week	265	3.25(21.64)	266	11.18(20.92)	-6.13(-9.33 to -2.92)	0.0002	-0.36(-0.54 to -0.17)
3 months	259	5.84(22.40)	260	14.33(19.91)	-6.37(-9.59 to -3.14)	0.0001	-0.41(-0.62 to -0.20)
**PedsQL school functioning**							
1 week	265	5.46(17.82)	266	7.83(17.84)	-3.44(-6.04 to -0.84)	0.0096	-0.20(-0.36 to -0.05)
3 months	259	7.27(17.56)	260	13.74(16.77)	-7.52(-10.15 to -4.90)	0.0001	-0.48(-0.64 to -0.31)
**PedsQL psychosocial health**							
1 week	265	4.64(15.03)	266	10.05(14.88)	-5.48(-8.08 to -2.87)	<.0001	-0.37(-0.55 to -0.19)
3 months	259	7.50(14.91)	260	14.78(14.91)	-7.26(-9.88 to -4.64)	<.0001	-0.54(-0.74 to -0.35)
**PSYCHLOPS-Kids**							

Visit	Descriptive statistics				Mixed model analysis		
	Wait-list control		EASE intervention		Difference (95%CI)	p-value	Effect Size
	n	Mean (SD)	n	Mean (SD)			
1 week	265	-0.84(3.76)	266	-2.35(4.22)	1. 89(1.19 to 2.60)	<.0001	0.48(0.30 to 0.65)
3 months	259	-0.88(3.96)	260	-2.91(4.00)	2. 41(1.69 to 3.12)	<.0001	0. 64(0.45 to 0.83)
**APQ involvement**							
1 week	265	3.11(12.04)	266	4. 17(12.96)	-2.50(-5.32 to 0.32)	0.0820	-0.19(-0.40 to 0.02)
3 months	259	7.50(13.12)	260	9. 02 (14.24)	-2.78 (-5.61 to 0.05)	0.0546	-0.20(-0.40 to 0.00)
**APQ positive parenting**							
1 week	265	0.25(4.88)	266	1. 43(4.87)	-1.33(-2.24 to -0.41)	0.0045	-0.29(-0.48 to -0.09)
3 months	259	0.92(4.96)	260	1. 35(5.16)	-0.55(-1.47 to 0.37)	0.2383	-0.12(-0.32 to 0.08)
**APQ poor supervision**							
1 week	265	-1.73(5.49)	266	-1.59 (5.56)	-0.16 (-1.11 to 0.79)	0.7430	-0.03(-0.22 to 0.16)
3 months	259	-3.07(6.09)	260	-3.52 (5.95)	0. 39 (-0.56 to 1.35)	0.4196	0. 07 (-0.10 to 0.23)
**APQ inconsistent discipline**							
1 week	265	0.77(4.25)	266	0.44(4.18)	0. 45(-0.13 to 1.03)	0.1266	0.13(-0.04 to 0.30)
3 months	259	1.24(4.40)	260	0.29(4.44)	1. 07(0.48 to 1.65)	0.0004	0.32(0.14 to 0.49)
**APQ corporal punishment**							
1 week	265	-0.45(2.26)	266	-0.69(2.44)	-0.03(-0.46 to 0.40)	0.8944	-0.02(-0.26 to 0.22)
3 months	259,	1.82(3.05)	260	1. 45(3.14)	0. 11 (-0.32 to 0.55)	0.6111	0.04(-0.12 to 0.21)
**Caregivers' report secondary outcomes**							
**PedsQL – family impact**							
1 week	265	4.08(12.22)	266	5.55(12.50)	-0.26(-1.31 to 0.78)	0.6213	-0.04(-0.19 to 0.12)
3 months	259	4.18(13.27)	260	6.02(12.27)	-0.82(-1.88 to 0.24)	0.1289	-0.14(-0.33 to 0.04)
**APQ involvement**							
1 week	265	3.34(7.91)	266	6.20(6.76)	-2.71(-4.04 to -1.38)	<.0001	-0.37(-0.55 to -0.19)
3 months	259	3.72(8.24)	260	6.06(7.15)	-2.22(-3.50 to -0.88)	0.0012	-0.31(-0.50 to -0.12)
**APQ positive parenting**							
1 week	265	1.35(5.54)	266	2.25(4.82)	-1.10(-2.20 to -0.01)	0.0488	-0.23 (-0.47 to -0.00)
3 months	259	1.38(5.56)	260	1.95(4.55)	-0.77(-1.88 to 0.33)	0.1686	-0.16(-0.39 to 0.07)
**APQ poor supervision**							
1 week	265	-0.10(3.78)	266	-2.26(4.46)	0.99(0.10 to 1.88)	0.0300	0.28(0.03 to 0.54)
3 months	259	-1.24(3.95)	260	-2.86(4.58)	0.43(-0.46 to 1.33)	0.3428	0.13(-0.13 to 0.39)
**APQ inconsistent discipline**							
1 week	265	1.10(5.02)	266	-7.44(128.99)	0. 21 (-0.66 to 1.08)	0.6382	0. 06 (-0.18 to 0.29)
3 months	259	1.07(4.78)	260	-7.25(130.55)	-0.14(-1.02 to 0.73)	0.7461	-0.04 (-0.28 to 0.20)
**APQ corporal punishment**							
1 week	265	0.02(2.66)	266	-0.50(3.09)	0. 10 (-0.35 to 0.55)	0.6649	0. 04(-0.14 to 0.22)
3 months	259	0.11(2.77)	260	-0.42(2.97)	0. 12 (-0.34 to 0.57)	0.6125	0.05(-0.14 to 0.24)

PSC=Pediatric Symptom Checklist. RCADS=Revised Children Anxiety and Depression Scale. SPSI-R=Social Problem Solving inventory. PEPS=Perceived Emotional/Personal Support Scale. PHQ-9=Patient Health Questionnaire. SWEMWS=Short Warwick Edinburgh Mental Wellbeing Scale. PSYCHLOPS-Kids=Psychological Outcome Profile-Kids version. PedsQL=Pediatrics Quality of Life – Score. APQ=Allabama Parenting Questionnaire

Table S4: Summary statistics and results from mixed model analysis of primary and secondary outcomes (changes from baseline): covariate adjusted analysis where age, cluster, PSC included as covariates (Intention to treat population)

Visit	Descriptive statistics		Mixed model analysis		Effect size*
	Wait-list control (N=284)	EASE intervention (N=282)	Difference (95%CI)	p-value	
	Mean (SD)	Mean (SD)			
**Child reported outcomes**					
**Primary outcome**					
**PSC total score**					
Baseline	33.69(4.89)	32.69(4.34)			
1 week	-12.42(8.58)	-14.85(9.16)	2.49(0.71 to 4.28)	0.0063	0.27(0.08 to 0.47)
3 months	-13.34(8.98)	-16.38(8.65)	3.26(1.46 to 5.06)	0.0004	0.35(0.16 to 0.55)
**Child reported secondary outcomes**					
**PSC internalizing score**					
Baseline	5.37(1.75)	5.1(1.88)			
1 week	-2.22(2.53)	-2.56(2.48)	0.46(0.10 to 0.83)	0.0138	0.22(0.04 to 0.39)
3 months	-2.53(2.62)	-3.00(2.38)	0.58(0.21 to 0.96)	0.0022	0.29(0.10 to 0.47)
**PSC externalizing score**					
Baseline	4.85(2.37)	5.07(2.32)			
1 week	-1.36(2.50)	-2.10(2.81)	0.48(0.11 to 0.84)	0.0103	0.24(0.06 to 0.42)
3 months	-1.54(2.57)	-2.25(2.91)	0.51(0.15 to 0.88)	0.0061	0.24(0.07 to 0.42)
**PSC attention score**					
Baseline	5.64(1.69)	5.48(1.83)			
1 week	-2.21(2.39)	-2.54(2.36)	0.30(-0.07 to 0.67)	0.1092	0.15(-0.03 to 0.34)
3 months	-2.24(2.42)	-2.79(2.34)	0.55(0.17 to 0.92)	0.0042	0.26(0.08 to 0.44)
**RCADS total score**					
Baseline	64.44(12.97)	61.69(12.92)			
1 week	-15.01(17.26)	-17.09(17.77)	4.18(1.98 to 6.39)	0.0002	0.33(0.16 to 0.51)
3 months	-17.27(15.06)	-20.98(12.98)	5.72(3.49 to 7.94)	<.0001	0.51(0.31 to 0.71)
**RCADS anxiety score**					
Baseline	63.63(12.52)	60.81(12.76)			
1 week	-13.70(16.45)	-15.48(17.50)	3.99(1.91 to 6.07)	0.0002	0.34(0.16 to 0.51)
3 months	-15.95(14.39)	-19.19(12.67)	5.40(3.30 to 7.50)	<.0001	0.51(0.31 to 0.71)
**RCADS depression score**					
Baseline	62(13.46)	60.65(12.22)			
1 week	-14.22(17.70)	-16.45(16.37)	3.03(0.87 to 5.19)	0.0061	0.25(0.07 to 0.42)
3 months	-15.56(15.16)	-19.60(13.20)	4.62(2.43 to 6.80)	<.0001	0.43(0.23 to 0.63)
**Somatic symptoms score**					
Baseline	4.1(3.23)	3.54(2.71)			
1 week	-0.12(3.74)	-0.59(3.70)	0.85(0.23 to 1.46)	0.0074	0.27(0.07 to 0.46)

Visit	Descriptive statistics		Mixed model analysis		Effect size*
	Wait-list control (N=284)	EASE intervention (N=282)	Difference (95%CI)	p-value	
	Mean (SD)	Mean (SD)			
3 months	-0.34(3.83)	-1.23(3.50)	1.20(0.58 to 1.82)	0.0002	0.40(0.19 to 0.60)
****Positive orientation towards problem solving**					
Baseline	7.85(3.09)	7.34(2.79)			
1 week	0.35(3.97)	1.15(3.55)	-0.27(-0.80 to 0.25)	0.3085	-0.09(-0.28 to 0.09)
3 months	0.82(4.07)	1.35(3.84)	-0.01(-0.54 to 0.52)	0.9705	-0.00(-0.20 to 0.19)
****Negative orientation towards problem solving**					
Baseline	8.57(4.59)	8.23(3.9)			
1 week	-1.49(4.59)	-2.35(4.70)	1.08(0.38 to 1.77)	0.0025	0.26(0.09 to 0.43)
3 months	-1.56(5.02)	-2.44(4.79)	1.00(0.30 to 1.70)	0.0053	0.25(0.07 to 0.42)
****Rational orientation towards problem solving**					
Baseline	7.37(4.57)	6.83(3.76)			
1 week	0.43(5.15)	1.87(4.69)	-0.94(-1.69 to -0.20)	0.0135	-0.23(-0.41 to -0.05)
3 months	1.32(5.64)	2.35(5.30)	-0.53(-1.29 to 0.22)	0.1645	-0.13(-0.31 to 0.05)
****Impulsive/Carelessness style towards problem solving**					
Baseline	5.47(5.15)	5.51(4.28)			
1 week	0.71(5.63)	-0.77(5.44)	1.23(0.44 to 2.03)	0.0024	0.25(0.09 to 0.42)
3 months	0.63(5.76)	-0.02(5.80)	0.40(-0.40 to 1.21)	0.3242	0.08(-0.08 to 0.24)
****Avoidance style towards problem solving**					
Baseline	5.52(3.56)	6.1(3.39)			
1 week	-0.50(4.46)	-1.83(4.45)	0.62(0.02 to 1.21)	0.0429	0.18(0.01 to 0.35)
3 months	-0.64(4.67)	-2.27(4.45)	0.92(0.31 to 1.52)	0.0030	0.27(0.09 to 0.45)
**PEPS support from family**					
Baseline	15.86(11.12)	18.17(10.95)			
1 week	-1.36(6.07)	0.35(7.01)	-1.94(-3.15 to -0.73)	0.0018	-0.18(-0.29 to -0.07)
3 months	-0.98(6.55)	0.50(6.84)	-1.62(-2.83 to -0.40)	0.0095	-0.14(-0.25 to -0.03)
**PEPS support from non-family adults**					
Baseline	8.2(9.3)	6.92(8.17)			
1 week	-1.78(5.32)	-1.05(4.56)	-0.30(-1.19 to 0.60)	0.5157	-0.04(-0.15 to 0.07)
3 months	-1.75(5.71)	-1.00(4.95)	-0.33(-1.23 to 0.57)	0.4705	-0.04(-0.15 to 0.07)
**PEPS support from friends**					
Baseline	17.43(12.85)	16.66(11.12)			
1 week	-2.68(8.00)	-1.38(8.04)	-0.82(-2.45 to 0.80)	0.3216	-0.07(-0.20 to 0.07)
3 months	-2.30(9.16)	-1.17(9.26)	-0.59(-2.23 to 1.05)	0.4786	-0.05(-0.18 to 0.08)
**PHQ-9-A score**					
Baseline	7.28(5.05)	6.4(4.11)			
1 week	-1.44(5.37)	-2.52(4.39)	1.63(0.90 to 2.36)	<.0001	0.38(0.21 to 0.55)
3 months	-2.28(5.48)	-3.53(4.44)	1.68(0.95 to 2.42)	<.0001	0.42(0.23 to 0.60)

Visit	Descriptive statistics		Mixed model analysis		Effect size*
	Wait-list control (N=284)	EASE intervention (N=282)	Difference (95%CI)	p-value	
	Mean (SD)	Mean (SD)			
**SWEMWS score**					
Baseline	18.3(3.37)	18.87(3.23)			
1 week	0.78(4.04)	1.83(4.27)	-1.38(-2.11 to -0.65)	0.0002	-0.37(-0.56 to -0.17)
3 months	1.21(4.05)	2.24(4.45)	-1.35(-2.09 to -0.62)	0.0003	-0.36(-0.55 to -0.16)
**PedsQL total score**					
Baseline	75.58(13)	75.25(12.16)			
1 week	4.00(13.69)	8.95(13.67)	-4.23(-6.57 to -1.89)	0.0004	-0.32(-0.49 to -0.14)
3 months	5.80(13.98)	12.91(13.87)	-6.20(-8.56 to -3.84)	<.0001	-0.48(-0.67 to -0.30)
**PedsQL physical functioning**					
Baseline	82.57(14.68)	81.18(14.14)			
1 week	3.18(18.74)	7.82(18.15)	-3.04(-5.83 to -0.24)	0.0334	-0.20(-0.38 to -0.02)
3 months	2.93(19.98)	10.69(18.41)	-5.69(-8.51to -2.87)	<.0001	-0.35(-0.52 to -0.18)
**PedsQL emotional functioning**					
Baseline	66.12(18.7)	66.84(17.27)			
1 week	5.16(18.94)	11.11(19.50)	-5.40(-8.52 to -2.28)	0.0007	-0.30(-0.47 to -0.13)
3 months	9.39(19.96)	16.28(21.19)	-6.39(-9.54 to -3.25)	<.0001	-0.36(-0.54 to -0.18)
**PedsQL social functioning**					
Baseline	81.24(19.57)	79.49(18.37)			
1 week	3.25(21.64)	11.01(20.79)	-5.03(-8.12 to -1.93)	0.0015	-0.29(-0.47 to -0.11)
3 months	5.84(22.40)	14.19(19.85)	-5.26(-8.38 to -2.14)	0.0010	-0.34(-0.54 to -0.14)
**PedsQL school functioning**					
Baseline	68.23(16.53)	70.06(14.61)			
1 week	5.46(17.82)	7.98(17.80)	-3.28(-5.89 to -0.66)	0.0142	-0.19(-0.35 to -0.04)
3 months	7.27(17.56)	13.77(16.80)	-7.29(-9.93 to -4.65)	<.0001	-0.46(-0.63 to -0.30)
**PedsQL psychosocial health**					
Baseline	71.86(14.27)	72.11(12.97)			
1 week	4.64(15.03)	10.05(14.76)	-4.89(-7.41to -2.37)	0.0002	-0.33(-0.50 to -0.16)
3 months	7.50(14.91)	14.77(14.86)	-6.65(-9.19 to -4.10)	<.0001	-0.50(-0.69 to -0.31)
**PSYCHLOPS-Kids**					
Baseline	6.98(3.5)	6.36(3.54)			
1 week	-0.84(3.76)	-2.34(4.20)	1.73(1.04 to 2.41)	<.0001	0.44(0.26 to 0.61)
3 months	-0.88(3.96)	-2.92(4.01)	2.25(1.56 to 2.94)	<.0001	0.60(0.41 to 0.78)
**APQ involvement**					
Baseline	39.73(11.96)	42.35(11.88)			
1 week	3.11(12.04)	4.17(12.96)	-2.38(-5.19 to 0.42)	0.0954	-0.18(-0.39 to 0.03)
3 months	7.50(13.12)	9.02(14.24)	-2.66(-5.47 to 0.16)	0.0644	-0.19(-0.39 to 0.01)
**APQ positive parenting**					

Visit	Descriptive statistics		Mixed model analysis		Effect size*
	Wait-list control (N=284)	EASE intervention (N=282)	Difference (95%CI)	p-value	
	Mean (SD)	Mean (SD)			
Baseline	18.24(4.91)	18.56(4.55)			
1 week	0.25(4.88)	1.43(4.87)	-1.25(-2.18 to -0.33)	0.0081	-0.27(-0.47 to -0.07)
3 months	0.92(4.96)	1.35(5.16)	-0.48(-1.41 to 0.45)	0.3139	-0.10(-0.31 to 0.10)
**APQ poor supervision**					
Baseline	19.88(5.38)	20.06(5.08)			
1 week	-1.73(5.49)	-1.59(5.56)	-0.26(-1.22 to 0.69)	0.5918	-0.05(-0.24 to 0.14)
3 months	-3.07(6.09)	-3.52(5.95)	0.29(-0.68 to1.25)	0.5584	0.05(-0.11 to 0.21)
**APQ inconsistent discipline**					
Baseline	12.88(3.35)	12.71(3.26)			
1 week	0.77(4.25)	0.44(4.18)	0.44(-0.14 to 1.03)	0.1368	0.13(-0.04 to 0.30)
3 months	1.24(4.40)	0.29(4.44)	1.06(0.47 to 1.65)	0.0005	0.32(0.14 to 0.49)
**APQ corporal punishment**					
Baseline	5.55(2.16)	5.77(2)			
1 week	-0.45(2.26)	-0.69(2.44)	-0.07(-0.51 to 0.37)	0.7583	-0.04(-0.28 to 0.21)
3 months	1.82(3.05)	1.45(3.14)	0.07(-0.37 to 0.52)	0.7528	0.03(-0.14 to 0.19)
**Caregiver reported secondary outcomes**					
**PedsQL – family impact**					
Baseline	92.54(13.93)	91.29(13.04)			
1 week	4.08(12.22)	5.49(12.44)	-0.23(-1.28 to 0.82)	0.6668	0.03(-0.19 to 0.12)
3 months	4.18(13.27)	6.00(12.23)	-0.79(-1.85 to 0.28)	0.1475	-0.14(-0.32 to 0.05)
**APQ involvement**					
Baseline	26.68(7.93)	26.15(7.23)			
1 week	3.34(7.91)	6.19(6.74)	-2.82(-4.14 to -1.49)	<.0001	-0.39(-0.57 to -0.20)
3 months	3.72(8.24)	6.02(7.13)	-2.28(-3.61 to-0.94)	0.0009	-0.32(-0.50 to -0.13)
**APQ positive parenting**					
Baseline	19.87(4.83)	20.03(4.31)			
1 week	1.35(5.54)	2.27(4.79)	-1.20(-2.30 to -0.11)	0.0315	-0.26(-0.49 to -0.02)
3 months	1.38(5.56)	1.94(4.55)	-0.82(-1.92 to 0.28)	0.1443	-0.17(-0.40 to 0.06)
**APQ poor supervision**					
Baseline	13.26(3.59)	14.92(4.33)			
1 week	-0.10(3.78)	-2.20(4.47)	0.99(0.09 to1.88)	0.0302	0.28(0.03 to 0.54)
3 months	-1.24(3.95)	-2.85(4.57)	0.45(-0.45 to 1.34)	0.3259	0.13(-0.13 to 0.39)
**APQ inconsistent discipline**					
Baseline	13.43(3.82)	13.8(3.39)			
1 week	1.10(5.02)	-7.34(127.79)	0.20(-0.69 to 1.08)	0.6616	0.05(-0.18 to 0.29)
3 months	1.07(4.78)	-7.14(129.80)	-0.22(-1.11 to 0.67)	0.6252	-0.06(-0.30 to 0.18)
**APQ corporal punishment**					

**Visit**	**Descriptive statistics**		**Mixed model analysis**		**Effect size***
	**Wait-list control (N=284)**	**EASE intervention (N=282)**	**Difference (95%CI)**	**p-value**	
	**Mean (SD)**	**Mean (SD)**			
Baseline	6.77(2.58)	7.38(2.68)			
1 week	0.02(2.66)	-0.52(3.08)	0.08(-0.37 to 0.53)	0.7327	0.03(-0.15 to 0.21)
3 months	0.11(2.77)	-0.45(2.99)	0.10(-0.36 to 0.55)	0.6768	0.04(-0.15 to 0.23)

PSC=Pediatric Symptoms Checklist. RCADS=Revised Children Anxiety and Depression Scale. **= subscales of Social Problem-Solving Inventory - Revised Short Form. PEPS= Perceived Emotional/Personal Support Scale. PHQ-A=Patient Health Questionnaire – Adolescent version. SWEMWS= Short Warwick Edinburgh Mental Wellbeing Scale. PedsQL=Pediatric Quality of Life. PSYCHLOPS-Kids= Psychological Outcome Profile-Kids version. APQ= Alabama Parenting Questionnaire.

*Effect size was calculated using the difference in least square means between intervention and control arm from mixed model divided by the pooled SD at each visit.

Table S4.1: Summary statistics and results from mixed model analysis of primary and secondary outcomes (changes from baseline): covariate adjusted analysis where age, cluster, PSC included as covariates (per protocol analysis)

	Descriptive statistics				Mixed model analysis		
	Wait-list control		EASE intervention				
Visit	n	Mean (SD)	n	Mean (SD)	Difference (95%CI)	p-value	Effect Size
**Child reported outcome measure**							
**PSC total score**							
1 week	265	-12.42(8.58)	266	-14.78(9.19)	2.43(0.64 to 4.22)	0.0078	0.27(0.07 to 0.46)
3 months	259	-13.34(8.98)	260	-16.39(8.67)	3.26(1.46 to 5.06)	0.0004	0.35(0.16 to 0.55)
**PSC internalizing score**							
1 week	265	-2.22(2.53)	266	-2.57(2.50)	0.45(0.08 to 0.83)	0.0167	0.21(0.04 to 0.39)
3 months	259	-2.53(2.62)	260	-3.01(2.38)	0.59(0.21 to 0.96)	0.0022	0.29(0.10 to 0.47)
**PSC externalizing score**							
1 week	265	-1.36(2.50)	266	-2.11(2.83)	0.48(0.11 to 0.84)	0.0106	0.24(0.06 to 0.42)
3 months	259	-1.54(2.57)	260	-2.26(2.92)	0.52(0.16 to 0.89)	0.0054	0.25(0.07 to 0.42)
**PSC attention score**							
1 week	265	-2.21(2.39)	266	-2.53(2.37)	0.29(-0.08 to 0.66)	0.1247	0.15(-0.04 to 0.33)
3 months	259	-2.24(2.42)	260	-2.78(2.34)	0.54(0.16 to 0.91)	0.0050	0.25(0.08 to 0.43)
**RCADS total score**							
1 week	265	-15.01(17.26)	266	-17.09(17.82)	4.19(1.98 to 6.41)	0.0002	0.33(0.16 to 0.51)
3 months	259	-17.27(15.06)	260	-20.90(12.99)	5.67(3.44 to 7.91)	<.0001	0.51(0.31 to 0.71)
**RCADS anxiety score**							
1 week	265	-13.70(16.45)	266	-15.46(17.52)	4.01(1.92 to 6.10)	0.0002	0.34(0.16 to 0.52)
3 months	259	-15.95(14.39)	260	-19.08(12.66)	5.36(3.25 to 7.47)	<.0001	0.50(0.30 to 0.70)
**RCADS depression score**							
1 week	265	-14.22(17.70)	266	-16.50(16.48)	3.00(0.83 to 5.17)	0.0069	0.24(0.07 to 0.42)
3 months	259	-15.56(15.16)	260	-19.61(13.23)	4.59(2.40 to 6.78)	<.0001	0.42(0.22 to 0.63)
**Somatic symptoms**							
1 week	265	-0.12(3.74)	266	-0.61(3.71)	0.85(0.23 to 1.47)	0.0071	0.27(0.07 to 0.47)
3 months	259	-0.34(3.83)	260	-1.23(3.52)	1.19(0.56 to 1.81)	0.0002	0.39(0.19 to 0.60)
****Positive orientation towards problem solving**							
1 week	265	0.35(3.97)	266	1.12(3.57)	-0.30(-0.83 to 0.24)	0.2776	-0.10(-0.29 to -0.08)
3 months	259	0.82(4.07)	260	1.28(3.78)	0.03(-0.51 to 0.57)	0.9221	0.01(-0.19 to 0.21)
****Negative orientation towards problem solving**							
1 week	265	265,-1.49(4.59)	266	-2.35(4.70)	1.06(0.36 to 1.76)	0.0030	0.26(0.09 to 0.43)
3 months	259	259,-1.56(5.02)	260	-2.46(4.81)	1.02(0.31 to 1.72)	0.0047	0.25(0.08 to 0.42)
****Rational orientation towards problem solving**							
1 week	265	0.43(5.15)	266	1.85(4.68)	-0.95(-1.71 to -0.19)	0.0145	-0.23(-0.41 to -0.05)
3 months	259	1.32(5.64)	260	2.25(5.25)	-0.47(-1.24 to 0.29)	0.2238	-0.11(-0.29 to 0.07)
****Impulsive/Carelessness problem solving style**							

Visit	Descriptive statistics				Mixed model analysis		
	Wait-list control		EASE intervention				
	n	Mean (SD)	n	Mean (SD)	Difference (95%CI)	p-value	Effect Size
1 week	265	0.71(5.63)	266	-0.82(5.47)	1.23(0.43 to 2.03)	0.0026	0.25(0.09 to 0.42)
3 months	259	0.63(5.76)	260	-0.04(5.81)	0.39(-0.41 to 1.20)	0.3371	0.08(-0.08 to 0.24)
****Avoidance problem solving style**							
1 week	265	-0.50(4.46)	266	-1.81(4.47)	0.57(-0.03 to 1.16)	0.0610	0.16(-0.01 to 0.33)
3 months	259	-0.64(4.67)	260	-2.29(4.47)	0.91(0.31 to 1.51)	0.0029	0.27(0.09 to 0.45)
**PEPSQ support from family**							
1 week	265	-1.36(6.07)	266	0.50(6.93)	-2.05(-3.24 to -0.86)	0.0008	-0.19(-0.30 to -0.08)
3 months	259	-0.98(6.55)	260	0.51(6.85)	-1.61(-2.80 to -0.41)	0.0085	-0.14(-0.25 to -0.04)
**PEPSQ support from non-family**							
1 week	265	-1.78(5.32)	266	-1.00(4.51)	-0.32(-1.21 to 0.56)	0.4742	-0.04(-0.15 to -0.07)
3 months	259	-1.75(5.71)	260	-0.96(4.95)	-0.39(-1.28 to 0.51)	0.3967	-0.05(-0.15 to -0.06)
**PEPSQ support from friends**							
1 week	265	-2.68(8.00)	266	-1.34(8.05)	-0.85(-2.48 to 0.78)	0.3077	-0.07(-0.21 to 0.07)
3 months	259	-2.30(9.16)	260	-1.13(9.29)	-0.66(-2.30 to 0.98)	0.4315	-0.05(-0.18 to 0.08)
**PHQ-9-A score**							
1 week	265	-1.44(5.37)	266	-2.56(4.42)	1.62(0.89 to 2.35)	<.0001	0.37(0.21 to 0.54)
3 months	259	-2.28(5.48)	260	-3.55(4.46)	1.68(0.94 to 2.42)	<.0001	0.41(0.23 to 0.60)
**SWEMWS score**							
1 week	265	1.08(5.60)	266	2.38(5.78)	-1.82(-2.80 to -0.85)	0.0003	-0.36(-0.55 to -0.17)
3 months	259	1.71(5.68)	260	2.95(5.92)	-1.73(-2.71to -0.75)	0.0006	-0.35(-0.55 to -0.16)
**PedsQL total score**							
1 week	265	4.00(13.69)	266	8.97(13.79)	-4.15(-6.50 to -1.80)	0.0006	-0.31(-0.49 to -0.13)
3 months	259	5.80(13.98)	260	12.97(13.91)	-6.17(-8.54 to -3.80)	<.0001	-0.48(-0.67 to -0.30)
**PedsQL physical functioning**							
1 week	265	3.18(18.74)	266	7.89(18.28)	-2.96(-5.78 to -0.15)	0.0391	-0.19(-0.37 to -0.01)
3 months	259	2.93(19.98)	260	10.85(18.43)	-5.71(-8.55 to -2.88)	<.0001	-0.35-(0.53 to -0.18)
**PedsQL emotional functioning**							
1 week	265	5.16(18.94)	266	11.08(19.67)	-5.31(-8.44 to -2.18)	0.0009	-0.29(-0.46 to -0.12)
3 months	259	9.39(19.96)	260	16.20(21.26)	-6.30(-9.45 to -3.14)	<.0001	-0.36(-0.54 to -0.18)
**PedsQL social functioning**							
1 week	265	3.25(21.64)	266	11.18(20.92)	-4.99(-8.09 to -1.88)	0.0017	-0.29(-0.47 to -0.11)
3 months	259	5.84(22.40)	260	14.33(19.91)	-5.25(-8.38 to -2.13)	0.0010	-0.34(-0.54 to -0.14)
**PedsQL school functioning**							
1 week	265	5.46(17.82)	266	7.83(17.84)	-3.11(-5.73 to -0.49)	0.0203	-0.18(-0.34 to -0.03)
3 months	259	7.27(17.56)	260	13.74(16.77)	-7.19(-9.84 to -4.55)	<.0001	-0.46(-0.63 to -0.29)
**PedsQL psychosocial health**							
1 week	265	4.64(15.03)	266	10.05(14.88)	-4.80(-7.33 to -2.27)	0.0002	-0.32(-0.50 to -0.15)
3 months	259	7.50(14.91)	260	14.78(14.91)	-6.59(-9.14 to -4.04)	<.0001	-0.49(-0.68 to -0.30)

	Descriptive statistics				Mixed model analysis		
	Wait-list control		EASE intervention				
Visit	n	Mean (SD)	n	Mean (SD)	Difference (95%CI)	p-value	Effect Size
**PSYCHLOPS-Kid**							
1 week	265	-0.84(3.76)	266	-2.35(4.22)	1.72(1.03 to 2.41)	<.0001	0.43(0.26 to 0.61)
3 months	259	-0.88(3.96)	260	-2.91(4.00)	2.24(1.54 to 2.93)	<.0001	0.59(0.41 to 0.78)
**APQ involvement**							
1 week	265	3.11(12.04)	266	4.24(13.02)	-2.40(-5.21 to 0.42)	0.0952	-0.18(-0.40 to 0.03)
3 months	259	7.50(13.12)	260	9.23(14.18)	-2.78(-5.61 to 0.04)	0.0535	-0.20(-0.39 to 0.00)
**APQ positive parenting**							
1 week	265	0.25(4.88)	266	1.44(4.89)	-1.25(-2.17 to -0.33)	0.0078	-0.27(-0.47 to -0.07)
3 months	259	0.92(4.96)	260	1.38(5.17)	-0.50(-1.42 to 0.43)	0.2923	-0.11(-0.31 to 0.09)
**APQ poor supervision**							
1 week	265	-1.73(5.49)	266	-1.63(5.59)	-0.26(-1.21 to 0.70)	0.5966	-0.05(-0.24 to 0.14)
3 months	259	-3.07(6.09)	260	-3.53(5.98)	0.27(-0.69 to 1.23)	0.5773	0.05(-0.12 to -0.21)
**APQ inconsistent discipline**							
1 week	265	0.77(4.25)	266	0.44(4.21)	0.43(-0.15 to 1.02)	0.1438	0.13(-0.04 to 0.30)
3 months	259	1.24(4.40)	260	0.29(4.44)	1.06(0.47 to 1.65)	0.0004	0.32(0.14 to 0.49)
**APQ corporal punishment**							
1 week	265	-0.45(2.26)	266	-0.70(2.44)	-0.06(-0.50 to 0.38)	0.7900	-0.03(-0.28 to 0.21)
3 months	259	1.82(3.05)	260	1.44(3.15)	0.07(-0.37 to 0.52)	0.7428	0.03(-0.14 to 0.20)
**Caregivers reported secondary outcomes**							
**PedsQL – family impact**							
1 week	265	4.08(12.22)	266	5.55(12.50)	-0.23(-1.29 to 0.83)	0.6717	-0.03(-0.19 to 0.12)
3 months	259	4.18(13.27)	260	6.02(12.27)	-0.79(-1.86 to 0.28)	0.1482	-0.14(-0.32 to 0.05)
**APQ involvement**							
1 week	265	3.34(7.91)	266	6.20(6.76)	-2.80(-4.13 to -1.46)	<.0001	-0.38(-0.57 to -0.20)
3 months	259	3.72(8.24)	260	6.06(7.15)	-2.29(-3.64 to -0.95)	0.0009	-0.32(-0.51 to -0.13)
**APQ positive parenting**							
1 week	265	1.35(5.54)	266	2.25(4.82)	-1.18(-2.28 to -0.08)	0.0353	-0.25(-0.48 to -0.02)
3 months	259	1.38(5.56)	260	1.95(4.55)	-0.84(-1.95 to 0.26)	0.1334	-0.18(-0.41 to 0.05)
**APQ poor supervision**							
1 week	265	-0.10(3.78)	266	-2.26(4.46)	1.01(0.11 to 1.91)	0.0273	0.29(0.03 to 0.55)
3 months	259	-1.24(3.95)	260	-2.86(4.58)	0.45(-0.45 to 1.35)	0.3232	0.13(-0.13 to 0.39)
**APQ inconsistent discipline**							
1 week	265	1.10(5.02)	266	-7.44(128.99)	0.14(-0.74 to 1.02)	0.7620	0.04(-0.20 to 0.27)
3 months	259	1.07(4.78)	260	-7.25(130.55)	-0.21(-1.09 to 0.68)	0.6452	-0.06(-0.30 to 0.19)
**APQ corporal punishment**							
1 week	265	0.02(2.66)	266	-0.50(3.09)	0.06(-0.39 to 0.52)	0.7807	0.03(-0.16 to 0.21)
3 months	259	0.11(2.77)	260	-0.42(2.97)	0.08(-0.37 to 0.54)	0.7162	0.04(-0.16 to 0.23)

PSC=Pediatric Symptoms Checklist (PSC). RCADS=Revised Children Anxiety and Depression Scale. PEPS=Perceived Emotional/Personal Support Questionnaire. PHQ-A=Patient Health Questionnaire – Adolescent version. SWEMWS=Short Warwick Edinburgh Mental Wellbeing

Scale. PedsQL=Pediatric Quality of Life. PSYCHLOPS-Kids=Psychological Outcome Profile-Kids version. APQ=Alabama Parenting Questionnaire

Table 5: Summary statistics and results from mixed model analysis of child reposted self-stigmatizing scale (Intention to treat population)

		Descriptive statistics n, mean (SD)		Unadjusted mixed model*		Adjusted mixed model	
**Measurement**	**Visit**	**Wait list Control**	**EASE intervention**	**Difference (95%CI)**	**p-value**	**Difference (95%CI)**	**p-value**
Self-Stigmatization Scale	3 months	259,9.75(0.78)	263,9.76(0.80)	0.00 (-0.19 to 0.19)	0.9741	0.01(-0.18 to 0.20)	0.8963

Table S5.1: Summary statistics and results from mixed model analysis of child reposted self-stigmatizing scale (per protocol analysis)

	Wait-list control		EASE intervention		Mixed model analysis	
**Visit**	**n**	**Mean (SD)**	**n**	**Mean (SD)**	**Difference (95%CI)**	**p-value**
Self-Stigmatization Scale						
3 months	259	9.75(0.78)	260	9.76(0.80)	0.01(-0.19 to 0.20)	0.9537

Table S6. Education/Health services utilization across two arms 3-months post intervention.

**Service providers accessed**	**Wait-list Control (*f*)**	**EASE intervention (*f*)**
**Services Utilized**	**85**	**85**
**Education Services**		
Home Tuition	12	6
Tuition Centre	73	76
Group Study	0	1
**Health Services**		
PHC	18	7
Hospital (Walk-in)	7	11
Private Doctor	44	55
Emergency	0	0
Homeopath	0	0
Traditional Healer	4	2
Child Mental Health	0	0
Other Services	0	0
Hospital Admission	0	0
Medicine Use	50	51
**Number of Health services visits**		
3+ visits	5	6
3 visits	9	8
2 visits	15	19
1 visit	37	38
**Medications**		
A. Anti-Medicine (n=16)		
- Anti-Depressants	1	0
- Anti-Infection	0	1
- Anti-Fungal	0	0
- Anti-Biotics	9	6
- Anti-Malarial	0	0
B. Allergy	7	9
C. ENT	8	10
D. Fever, Painkiller, Flu, Paracetamol	53	64
E. Multi-vitamins, Vaccine	2	5
F. Diarrhea	4	2
G. Others (anti- hypertensive, Ointment, deworming, Stomach, Vomit, bedwetting, pimples, thyroid related)	10	15
**Average duration of consultation (in minutes) M(SD); min-max**	15.79 (±25.72); 5-180	11.56 (±11.97); 2-90
**Average consultation fee paid (in PKR) M(SD); min-max**	601.52(±880); 0-4000	1221.27 (±5905.34); 0-50000

PHC= Primary healthcare. M=Mean. SD= Standard Deviation. PKR= Pakistani Rupees.

Table S7a: Summary statistics and results from mixed model analysis of primary and secondary outcomes (changes from baseline): Sensitivity analysis (multiple imputation) – Intension to Treat (ITT) Population

				Mixed model analysis		
	Visit	Treatment A (N=284)	Treatment B (N=282)	Difference (95%CI)	p-value	Effect Size
**Primary outcomes**						
PSC Full Scale Score	1 week	284, -12.58(8.36)	282, -14.72(9.02)	2.74(0.94 to 4.55)	0.0029	0.30(0.10 to 0.50)
	3 months	284, -13.57(8.70)	282, -16.29(8.46)	3.48(1.6 to 5.29)	0.0002	0.38(0.18 to 0.58)
**Child reported secondary outcomes**						
PSC internalizing score	1 week	284, -2.19(2.47)	282, -2.55(2.45)	0.58(0.19 to 0.98)	0.0042	0.27(0.09 to 0.46)
	3 months	284, -2.47(2.57)	282, -2.95(2.33)	0.70(0.30 to 1.10)	0.0007	0.34(0.15 to 0.54)
PSC externalizing score	1 week	284, -1.37(2.45)	282, -2.10(2.77)	0.57(0.19 to 0.95)	0.0035	0.29(0.10 to 0.48)
	3 months	284, -1.55(2.49)	282, -2.30(2.87)	0.61(0.22 to 0.99)	0.0021	0.29(0.11 to 0.47)
PSC attention score	1 week	284, -2.21(2.34)	282, -2.50(2.34)	0.44(0.07 to 0.82)	0.0214	0.23(0.03 to 0.42)
	3 months	284, -2.25(2.36)	282, -2.77(2.30)	0.68(0.30 to 1.06)	0.0005	0.32(0.14 to 0.50)
RCADS total score	1 week	284, -15.02(16.85)	282, -16.69(17.74)	4.31(2.11 to 6.52)	0.0001	0.35(0.17 to 0.52)
	3 months	284, -17.36(14.58)	282, -20.50(12.94)	5.82(3.59 to 8.05)	<.0001	0.52(0.32 to 0.72)
RCADS anxiety score	1 week	284, -13.63(16.02)	282, -15.08(17.43)	4.09(2.01 to 6.16)	0.0001	0.35(0.17 to 0.52)
	3 months	284, -16.01(13.86)	282, -18.75(12.60)	5.47(3.37 to 7.57)	<.0001	0.52(0.32 to 0.71)
RCADS depression score	1 week	284, -14.24(17.39)	282, -16.11(16.39)	3.35(1.17 to 5.53)	0.0027	0.27(0.09 to 0.45)
	3 months	284, -15.76(14.97)	282, -19.17(13.32)	4.91(2.70 to 7.11)	<.0001	0.45(0.25 to 0.66)
Somatic symptoms score	1 week	284, -0.09(3.67)	282, -0.55(3.67)	0.92(0.27 to 1.56)	0.0056	0.29(0.09 to 0.50)
	3 months	284, -0.40(3.72)	282, -1.13(3.46)	1.27(0.62 to 1.92)	0.0001	0.42(0.20 to 0.64)
**Positive orientation towards problem solving	1 week	284,0.56(1.70)	282,0.69(1.39)	-0.29(-0.81 to 0.24)	0.2828	-0.10(-0.28 to 0.08)
	3 months	284,0.91(2.83)	282,1.05(2.49)	-0.02(-0.55 to 0.51)	0.9362	-0.01(-0.21 to 0.19)
**Negative orientation towards problem solving	1 week	284, -1.97(1.88)	282, -1.68(1.90)	1.15(0.44 to 1.86)	0.0015	0.28(0.11 to 0.45)
	3 months	284, -1.55(5.57)	282, -2.32(5.15)	1.08(0.36 to 1.79)	0.0032	0.26(0.09 to 0.44)
**Rational orientation	1 week	284,1.13(2.07)	282,1.02(1.81)	-1.09(-1.82 to -0.35)	0.0038	-0.26(-0.44 to -0.09)

towards problem solving						
	3 months	284,1.45(4.39)	282,1.95(4.19)	-0.67(-1.42 to 0.07)	0.0764	-0.16(-0.34 to 0.02)
**Impulsive/Carelessness style towards problem solving	1 week	284,0.06(1.98)	282, -0.04(1.93)	1.47(0.65 to 2.29)	0.0004	0.30(0.13 to 0.47)
	3 months	284,0.22(4.97)	282, -0.15(4.87)	0.64(-0.19 to 1.47)	0.1321	0.13(-0.04 to 0.30)
**Avoidance style towards problem solving	1 week	284, -0.76(1.82)	282, -1.33(1.72)	0.80(0.21 to 1.38)	0.0081	0.23(0.06 to 0.40)
	3 months	284, -1.02(4.10)	282, -1.99(3.49)	1.09(0.49 to 1.68)	0.0004	0.32(0.15 to 0.50)
PEPS support from family	1 week	284, -1.24(5.92)	282,0.32(6.89)	-2.11(-3.39 to -0.83)	0.0013	-0.19(-0.31 to -0.08)
	3 months	284, -0.87(6.34)	282,0.35(6.69)	-1.78(-3.07 to -0.50)	0.0067	-0.16(-0.27 to -0.04)
PEPS support from non-family adults	1 week	284, -1.68(5.23)	282, -0.96(4.52)	-0.43(-1.37 to 0.50)	0.3640	-0.05(-0.17 to 0.06)
	3 months	284, -1.64(5.63)	282, -0.90(4.89)	-0.46(-1.40 to 0.49)	0.3405	-0.06(-0.17 to 0.06)
PEPS support from friends	1 week	284, -2.54(7.82)	282, -1.47(7.92)	-0.88(-2.50 to 0.74)	0.2852	-0.07(-0.21 to 0.06)
	3 months	284, -2.17(8.88)	282, -1.32(9.06)	-0.65(-2.28 to 0.99)	0.4375	-0.05(-0.18 to 0.08)
PHQ-9-A score	1 week	284, -1.43(5.23)	282, -2.51(4.34)	1.71(0.97 to 2.45)	<.0001	0.40(0.23 to 0.57)
	3 months	284, -2.33(5.32)	282, -3.46(4.37)	1.77(1.03 to 2.52)	<.0001	0.44(0.25 to 0.62)
SWEMWS score	1 week	284,0.50(4.29)	282,2.07(4.58)	-1.48(-2.22 to -0.74)	<.0001	-0.39(-0.59 to -0.20)
	3 months	284,1.55(1.46)	282,1.74(1.42)	-1.44(-2.18 to -0.70)	0.0001	-0.38(-0.58 to -0.19)
PedsQL total score	1 week	284,5.63(10.25)	282,7.49(10.62)	-4.86(-7.27 to -2.45)	<.0001	-0.36(-0.54 to -0.18)
	3 months	284,8.03(12.50)	282,10.42(12.59)	-6.84(-9.26 to -4.41)	<.0001	-0.53(-0.72 to -0.34)
PedsQL physical functioning	1 week	284,3.15(18.23)	282,7.96(18.14)	-3.90(-6.80 to -0.99)	0.0086	-0.25(-0.44 to -0.06)
	3 months	284,3.54(19.61)	282,10.58(18.31)	-6.55(-9.48 to -3.62)	<.0001	-0.40(-0.58 to -0.22)
PedsQL emotional functioning	1 week	284,5.29(18.46)	282,10.92(19.26)	-6.25(-9.46 to -3.04)	0.0001	-0.34(-0.52 to -0.17)
	3 months	284,9.52(19.36)	282,16.05(20.75)	-7.20(-10.44 to -3.96)	<.0001	-0.41(-0.60 to -0.23)
PedsQL social functioning	1 week	284,3.56(21.40)	282,10.62(20.55)	-6.20(-9.39 to -3.01)	0.0002	-0.36(-0.55 to -0.18)
	3 months	284,6.25(22.01)	282,13.52(19.55)	-6.39(-9.60 to -3.17)	0.0001	-0.41(-0.62 to -0.20)
PedsQL school functioning	1 week	284,5.42(17.48)	282,8.08(17.52)	-3.64(-6.24 to -1.05)	0.0060	-0.21(-0.37 to -0.06)
	3 months	284,7.50(17.13)	282,13.66(16.35)	-7.64(-10.27 to -5.02)	<.0001	-0.49(-0.65 to -0.32)
PedsQL psychosocial health	1 week	284,4.87(14.76)	282,9.83(14.59)	-5.58(-8.18 to -2.99)	<.0001	-0.38(-0.55 to -0.20)
	3 months	284,7.87(14.53)	282,14.49(14.49)	-7.33(-9.95 to -4.71)	<.0001	-0.55(-0.74 to -0.35)

PedsQL Physical Functioning Subscale Score	1 week	284,4.89(15.71)	282,4.98(15.18)	0.15(-1.40 to 1.71)	0.8451	0.02(-0.15 to 0.19)
	3 months	284,4.07(16.54)	282,5.45(14.84)	-1.29(-2.86 to 0.28)	0.1067	-0.15(-0.33 to 0.03)
PedsQL Emotional Functioning Subscale Score	1 week	284,4.65(18.09)	282,8.14(18.68)	-0.97(-3.53 to 1.59)	0.4564	-0.08(-0.28 to 0.12)
	3 months	284,4.36(19.90)	282,8.95(17.46)	-2.08(-4.66 to 0.51)	0.1147	-0.17(-0.38 to 0.04)
PedsQL Social Functioning Subscale Score	1 week	284,3.15(12.00)	282,3.19(11.04)	-0.18(-1.05 to 0.68)	0.6766	-0.04(-0.20 to 0.13)
	3 months	284,3.11(13.20)	282,3.22(12.16)	-0.21(-1.09 to 0.67)	0.6323	-0.04(-0.21 to 0.13)
PSYCHLOPS-Kids	1 week	284, -0.92(3.71)	282, -2.33(4.16)	1.92(1.21 to 2.62)	<.0001	0.48(0.31 to 0.66)
	3 months	284, -0.96(3.86)	282, -2.85(3.94)	2.43(1.72 to 3.14)	<.0001	0.65(0.46 to 0.83)
APQ involvement	1 week	284,4.73(12.77)	282,2.07(12.62)	-2.50(-5.32 to 0.32)	0.0820	-0.19(-0.41 to 0.02)
	3 months	284,9.07(12.42)	282,6.44(12.94)	-2.78(-5.61 to 0.05)	0.0546	-0.20(-0.40 to 0.00)
APQ positive parenting	1 week	284,0.28(4.81)	282,1.33(4.83)	-1.33(-2.24 to -0.41)	0.0045	-0.29(-0.48 to -0.09)
	3 months	284,0.92(4.85)	282,1.22(5.03)	-0.55(-1.47 to 0.37)	0.2383	-0.12(-0.32 to 0.08)
APQ poor supervision	1 week	284, -1.70(5.45)	282, -1.58(5.51)	-0.16(-1.11 to 0.79)	0.7430	-0.03(-0.22 to 0.16)
	3 months	284, -3.07(5.98)	282, -3.45(5.83)	0.39(-0.56 to 1.35)	0.4196	0.07(-0.10 to 0.23)
APQ inconsistent discipline	1 week	284,0.73(4.19)	282,0.45(4.14)	0.45(-0.13 to 1.03)	0.1266	0.13(-0.04 to 0.30)
	3 months	284,1.19(4.35)	282,0.34(4.32)	1.07(0.48 to 1.65)	0.0004	0.32(0.14 to 0.49)
APQ corporal punishment	1 week	284, -0.63(2.36)	282, -0.74(2.44)	-0.03(-0.46 to 0.40)	0.8944	-0.02(-0.26 to 0.22)
	3 months	284,1.64(3.09)	282,1.39(3.10)	0.11(-0.32 to 0.55)	0.6111	0.04(-0.12 to 0.21)
**Caregiver reported secondary outcomes**						
PedsQL – family impact	1 week	284,3.90(13.27)	282,5.41(12.29)	-0.27(-1.31 to 0.77)	0.6131	-0.04(-0.19 to 0.11)
	3 months	284,4.38(13.14)	282,5.53(12.48)	-0.82(-1.87 to 0.23)	0.1274	-0.14(-0.33 to 0.04)
APQ involvement (Caregiver)	1 week	284,3.44(7.69)	282,6.15(6.66)	-2.73(-4.05 to -1.41)	<.0001	-0.37(-0.56 to -0.19)
	3 months	284,3.79(7.99)	282,6.01(6.95)	-2.20(-3.53 to -0.87)	0.0012	-0.31(-0.49 to -0.12)
APQ positive parenting (Caregiver)	1 week	284,1.32(5.41)	282,2.22(4.74)	-1.13(-2.22 to -0.03)	0.0442	-0.24(-0.47 to -0.01)
	3 months	284,1.37(5.37)	282,1.92(4.50)	-0.75(-1.85 to 0.35)	0.1822	-0.16(-0.39 to 0.07)
APQ poor supervision (Caregiver)	1 week	284, -0.15(3.77)	282, -2.11(4.42)	0.96(0.08 to 1.85)	0.0332	0.28(0.02 to 0.53)
	3 months	284, -1.25(3.92)	282, -2.68(4.49)	0.43(-0.46 to 1.32)	0.3456	0.12(-0.13 to 0.38)

APQ inconsistent discipline (Caregiver)	1 week	284,1.12(4.98)	282, -7.01(125.28)	0.28(-0.59 to 1.15)	0.5335	0.07(-0.16 to 0.31)
	3 months	284,1.13(4.73)	282, -6.58(125.36)	-0.15(-1.03 to 0.72)	0.7316	-0.04(-0.28 to 0.20)
APQ corporal punishment (Caregiver)	1 week	284,0.02(2.59)	282, -0.52(3.06)	0.12(-0.33 to 0.56)	0.6096	0.05(-0.13 to 0.23)
	3 months	284,0.12(2.68)	282, -0.41(2.94)	0.13(-0.32 to 0.58)	0.5665	0.05(-0.13 to 0.24)

PSC=Pediatric Symptoms Checklist (PSC). RCADS=Revised Children Anxiety and Depression Scale. **= subscales of Social Problem-Solving Inventory - Revised Short Form. PEPS= Perceived Emotional/Personal Support Scale. PHQ-A=Patient Health Questionnaire – Adolescent version. SWEMWS= Short Warwick Edinburgh Mental Wellbeing Scale. PedsQL=Pediatric Quality of Life. PSYCHLOPS-Kids= Psychological Outcome Profile-Kids version. APQ=Alabama Parenting Questionnaire (APQ).

Table S7b: Summary statistics and results from mixed model analysis of primary and secondary outcomes (changes from baseline): Sensitivity analysis (multiple imputation) – Per Protocol Analysis

				**Mixed model analysis**		
	**Visit**	**Treatment A (N=284)**	**Treatment B (N=282)**	**Difference (95%CI)**	**p-value**	**Effect Size**
**Primary outcomes**						
PSC Full Scale Score	1 week	284, -12.58(8.36)	274, -14.67(9.10)	2.65(0.85 to 4.46)	0.0040	0.29(0.09 to 0.49)
	3 months	284, -13.57(8.70)	274, -16.32(8.54)	3.45(1.64 to 5.27)	0.0002	0.37(0.18 to 0.57)
**Child reported secondary outcomes**						
PSC internalizing score	1 week	284, -2.19(2.47)	274, -2.55(2.47)	0.57(0.17 to 0.96)	0.0051	0.27(0.08 to 0.45)
	3 months	284, -2.47(2.57)	274, -2.97(2.34)	0.70(0.30 to 1.10)	0.0007	0.34(0.15 to 0.54)
PSC externalizing score	1 week	284, -1.37(2.45)	274, -2.13(2.79)	0.57(0.19 to 0.95)	0.0037	0.29(0.09 to 0.48)
	3 months	284, -1.55(2.49)	274, -2.34(2.89)	0.62(0.23 to 1.00)	0.0019	0.29(0.11 to 0.48)
PSC attention score	1 week	284, -2.21(2.34)	274, -2.49(2.36)	0.42(0.05 to 0.80)	0.0277	0.22(0.02 to 0.41)
	3 months	284, -2.25(2.36)	274, -2.77(2.32)	0.67(0.28 to 1.05)	0.0006	0.31(0.13 to 0.49)
RCADS total score	1 week	284, -15.02(16.85)	274, -16.82(17.84)	4.31(2.09 to 6.52)	0.0001	0.34(0.17 to 0.52)
	3 months	284, -17.36(14.58)	274, -20.53(13.02)	5.76(3.53 to 7.99)	<.0001	0.52(0.32 to 0.72)
RCADS anxiety score	1 week	284, -13.63(16.02)	274, -15.14(17.53)	4.09(2.01 to 6.18)	0.0001	0.35(0.17 to 0.52)
	3 months	284, -16.01(13.86)	274, -18.75(12.65)	5.41(3.31 to 7.52)	<.0001	0.51(0.31 to 0.71)
RCADS depression score	1 week	284, -14.24(17.39)	274, -16.26(16.53)	3.30(1.11 to 5.49)	0.0032	0.27(0.09 to 0.44)
	3 months	284, -15.76(14.97)	274, -19.31(13.41)	4.87(2.66 to 7.08)	<.0001	0.45(0.25 to 0.66)
Somatic symptoms score	1 week	284, -0.09(3.67)	274, -0.59(3.70)	0.92(0.27 to 1.57)	0.0056	0.29(0.09 to 0.50)
	3 months	284, -0.40(3.72)	274, -1.15(3.49)	1.25(0.60 to 1.90)	0.0002	0.41(0.20 to 0.63)
**Positive orientation towards problem solving	1 week	284,0.56(1.70)	274,0.69(1.36)	-0.31(-0.84 to 0.22)	0.2548	-0.11(-0.29 to 0.08)
	3 months	284,0.91(2.83)	274,1.01(2.46)	0.02(-0.52 to 0.55)	0.9563	0.01(-0.20 to 0.21)
**Negative orientation towards problem solving	1 week	284, -1.97(1.88)	274, -1.69(1.85)	1.13(0.42 to 1.84)	0.0018	0.28(0.10 to 0.45)
	3 months	284, -1.55(5.57)	274, -2.39(5.18)	1.09(0.37 to 1.81)	0.0029	0.27(0.09 to 0.44)
**Rational orientation towards problem solving	1 week	284,1.13(2.07)	274,1.04(1.79)	-1.09(-1.84 to -0.34)	0.0045	-0.26(-0.45 to -0.08)
	3 months	284,1.45(4.39)	274,1.86(4.19)	-0.61(-1.36 to 0.15)	0.1136	-0.15(-0.33 to 0.03)

**Impulsive/Carelessness style towards problem solving	1 week	284,0.06(1.98)	274, -0.04(1.89)	1.47(0.64 to 2.29)	0.0005	0.30(0.13 to 0.47)
	3 months	284,0.22(4.97)	274, -0.15(4.90)	0.62(-0.21 to 1.46)	0.1414	0.13(-0.04 to 0.29)
**Avoidance style towards problem solving	1 week	284, -0.76(1.82)	274, -1.34(1.68)	0.74(0.15 to 1.33)	0.0135	0.21(0.04 to 0.38)
	3 months	284, -1.02(4.10)	274, -2.03(3.51)	1.08(0.49 to 1.67)	0.0004	0.32(0.14 to 0.50)
PEPS support from family	1 week	284, -1.24(5.92)	274,0.48(6.84)	-2.22(-3.48 to -0.96)	0.0006	-0.20(-0.32 to -0.09)
	3 months	284, -0.87(6.34)	274,0.41(6.73)	-1.78(-3.04 to -0.51)	0.0060	-0.16(-0.27 to -0.04)
PEPS support from non-family adults	1 week	284, -1.68(5.23)	274, -0.95(4.47)	-0.45(-1.39 to 0.48)	0.3406	-0.06(-0.17 to 0.06)
	3 months	284, -1.64(5.63)	274, -0.86(4.87)	-0.51(-1.45 to 0.43)	0.2896	-0.06(-0.17 to 0.05)
PEPS support from friends	1 week	284, -2.54(7.82)	274, -1.45(7.98)	-0.91(-2.53 to 0.72)	0.2735	-0.08(-0.21 to 0.06)
	3 months	284, -2.17(8.88)	274, -1.27(9.13)	-0.71(-2.35 to 0.93)	0.3940	-0.06(-0.18 to 0.07)
PHQ-9-A score	1 week	284, -1.43(5.23)	274, -2.55(4.38)	1.71(0.96 to 2.45)	<.0001	0.40(0.22 to 0.57)
	3 months	284, -2.33(5.32)	274, -3.52(4.40)	1.77(1.02 to 2.52)	<.0001	0.44(0.25 to 0.62)
SWEMWS score	1 week	284,0.50(4.29)	274,2.08(4.56)	-1.44(-2.19 to -0.70)	0.0001	-0.38(-0.58 to -0.19)
	3 months	284,1.55(1.46)	274,1.76(1.41)	-1.41(-2.16 to -0.66)	0.0002	-0.37(-0.57 to -0.18)
PedsQL total score	1 week	284,5.63(10.25)	274,7.64(10.69)	-4.77(-7.19 to -2.35)	0.0001	-0.36(-0.54 to -0.18)
	3 months	284,8.03(12.50)	274,10.57(12.71)	-6.80(-9.24 to -4.37)	<.0001	-0.53(-0.72 to -0.34)
PedsQL physical functioning	1 week	284,3.15(18.23)	274,8.02(18.35)	-3.81(-6.72 to -0.89)	0.0107	-0.25(-0.43 to -0.06)
	3 months	284,3.54(19.61)	274,10.74(18.44)	-6.57(-9.51 to -3.63)	<.0001	-0.40(-0.59 to -0.22)
PedsQL emotional functioning	1 week	284,5.29(18.46)	274,10.86(19.51)	-6.14(-9.36 to -2.91)	0.0002	-0.34(-0.51 to -0.16)
	3 months	284,9.52(19.36)	274,16.04(20.96)	-7.08(-10.33 to -3.84)	<.0001	-0.40(-0.59 to -0.22)
PedsQL social functioning	1 week	284,3.56(21.40)	274,10.89(20.76)	-6.13(-9.33 to -2.92)	0.0002	-0.36(-0.54 to -0.17)
	3 months	284,6.25(22.01)	274,13.83(19.71)	-6.37(-9.59 to -3.14)	0.0001	-0.41(-0.62 to -0.20)
PedsQL school functioning	1 week	284,5.42(17.48)	274,7.90(17.63)	-3.44(-6.04 to -0.84)	0.0096	-0.20(-0.36 to -0.05)
	3 months	284,7.50(17.13)	274,13.67(16.44)	-7.52(-10.15 to -4.90)	<.0001	-0.48(-0.65 to -0.31)
PedsQL psychosocial health	1 week	284,4.87(14.76)	274,9.91(14.73)	-5.48(-8.08 to -2.87)	<.0001	-0.37(-0.55 to -0.19)
	3 months	284,7.87(14.53)	274,14.58(14.64)	-7.26(-9.88 to -4.64)	<.0001	-0.54(-0.74 to -0.35)
PedsQL Physical Functioning Subscale Score	1 week	284,4.89(15.71)	274,4.98(15.17)	0.16(-1.40 to 1.73)	0.8364	0.02(-0.15 to 0.19)
	3 months	284,4.07(16.54)	274,5.46(14.87)	-1.27(-2.85 to 0.31)	0.1141	-0.15(-0.33 to 0.04)

PedsQL Emotional Functioning Subscale Score	1 week	284,4.65(18.09)	274,8.31(18.72)	-0.98(-3.54 to 1.58)	0.4534	-0.08(-0.28 to 0.12)
	3 months	284,4.36(19.90)	274,9.14(17.50)	-2.10(-4.69 to 0.48)	0.1098	-0.17(-0.38 to 0.04)
PedsQL Social Functioning Subscale Score	1 week	284,3.15(12.00)	274,3.22(11.17)	-0.17(-1.05 to 0.70)	0.6957	-0.03(-0.20 to 0.13)
	3 months	284,3.11(13.20)	274,3.27(12.31)	-0.21(-1.09 to 0.68)	0.6448	-0.04(-0.21 to 0.13)
PSYCHLOPS-Kids	1 week	284, -0.92(3.71)	274, -2.34(4.20)	1.89(1.19 to 2.60)	<.0001	0.48(0.30 to 0.66)
	3 months	284, -0.96(3.86)	274, -2.87(3.96)	2.41(1.69 to 3.12)	<.0001	0.64(0.45 to 0.83)
APQ involvement	1 week	284,4.73(12.77)	274,2.18(12.66)	-2.52(-5.35 to 0.31)	0.0808	-0.19(-0.41 to 0.02)
	3 months	284,9.07(12.42)	274,6.64(12.94)	-2.91(-5.75 to -0.07)	0.0443	-0.21(-0.41 to -0.01)
APQ positive parenting	1 week	284,0.28(4.81)	274,1.36(4.86)	-1.33(-2.24 to -0.42)	0.0043	-0.29(-0.48 to -0.09)
	3 months	284,0.92(4.85)	274,1.26(5.08)	-0.57(-1.49 to 0.34)	0.2186	-0.13(-0.33 to 0.07)
APQ poor supervision	1 week	284, -1.70(5.45)	274, -1.63(5.56)	-0.15(-1.10 to 0.80)	0.7495	-0.03(-0.22 to 0.16)
	3 months	284, -3.07(5.98)	274, -3.49(5.89)	0.38(-0.58 to 1.34)	0.4345	0.06(-0.10 to 0.23)
APQ inconsistent discipline	1 week	284,0.73(4.19)	274,0.42(4.17)	0.44(-0.14 to 1.02)	0.1339	0.13(-0.04 to 0.30)
	3 months	284,1.19(4.35)	274,0.29(4.34)	1.07(0.48 to 1.65)	0.0004	0.32(0.14 to 0.50)
APQ corporal punishment	1 week	284, -0.63(2.36)	274, -0.74(2.45)	-0.02(-0.46 to 0.41)	0.9273	-0.01(-0.25 to 0.23)
	3 months	284,1.64(3.09)	274,1.41(3.13)	0.12(-0.32 to 0.56)	0.6013	0.04(-0.12 to 0.21)
**Caregiver reported secondary outcomes**						
PedsQL – family impact	1 week	284,3.90(13.27)	274,5.45(12.39)	-0.26(-1.31 to 0.78)	0.6213	-0.04(-0.19 to 0.12)
	3 months	284,4.38(13.14)	274,5.56(12.59)	-0.82(-1.88 to 0.24)	0.1289	-0.14(-0.33 to 0.04)
APQ involvement (Caregiver)	1 week	284,3.44(7.69)	274,6.15(6.71)	-2.71(-4.04 to -1.38)	<.0001	-0.37(-0.56 to -0.19)
	3 months	284,3.79(7.99)	274,6.03(7.02)	-2.22(-3.56 to -0.88)	0.0012	-0.31(-0.50 to -0.12)
APQ positive parenting (Caregiver)	1 week	284,1.32(5.41)	274,2.23(4.78)	-1.10(-2.20 to -0.01)	0.0488	-0.24(-0.47 to -0.00)
	3 months	284,1.37(5.37)	274,1.95(4.52)	-0.77(-1.88 to 0.33)	0.1686	-0.16(-0.39 to 0.07)
APQ poor supervision (Caregiver)	1 week	284, -0.15(3.77)	274, -2.16(4.43)	0.99(0.10 to 1.88)	0.0300	0.28(0.03 to 0.54)
	3 months	284, -1.25(3.92)	274, -2.71(4.52)	0.43(-0.46 to 1.33)	0.3428	0.13(-0.13 to 0.39)
APQ inconsistent discipline (Caregiver)	1 week	284,1.12(4.98)	274, -7.17(127.09)	0.21(-0.66 to 1.08)	0.6382	0.06(-0.18 to 0.29)
	3 months	284,1.13(4.73)	274, -6.81(127.17)	-0.14(-1.02 to 0.73)	0.7461	-0.04(-0.28 to 0.20)

APQ corporal punishment (Caregiver)	1 week	284,0.02(2.59)	274, -0.49(3.06)	0.10(-0.35 to 0.55)	0.6649	0.04(-0.14 to 0.22)
	3 months	284,0.12(2.68)	274, -0.39(2.92)	0.12(-0.34 to 0.57)	0.6125	0.05(-0.14 to 0.24)

PSC=Pediatric Symptoms Checklist (PSC). RCADS=Revised Children Anxiety and Depression Scale. **= subscales of Social Problem-Solving Inventory - Revised Short Form. PEPS= Perceived Emotional/Personal Support Scale. PHQ-A=Patient Health Questionnaire – Adolescent version. SWEMWS= Short Warwick Edinburgh Mental Wellbeing Scale. PedsQL=Pediatric Quality of Life. PSYCHLOPS-Kids= Psychological Outcome Profile-Kids version. APQ=Alabama Parenting Questionnaire (APQ).
